# *Mahonia* vs. *Berberis* Unloaded: Generic Delimitation and Infrafamilial Classification of Berberidaceae Based on Plastid Phylogenomics

**DOI:** 10.3389/fpls.2021.720171

**Published:** 2022-01-06

**Authors:** Chia-Lun Hsieh, Chih-Chieh Yu, Yu-Lan Huang, Kuo-Fang Chung

**Affiliations:** ^1^Biodiversity Research Center, Academia Sinica, Taipei, Taiwan; ^2^School of Forestry and Resource Conservation, National Taiwan University, Taipei, Taiwan

**Keywords:** *accD* length variation, cytonuclear discordance, IR expansion, molecular dating, tribal classification

## Abstract

The early-diverging eudicot family Berberidaceae is composed of a morphologically diverse assemblage of disjunctly distributed genera long praised for their great horticultural and medicinal values. However, despite century-long studies, generic delimitation of Berberidaceae remains controversial and its tribal classification has never been formally proposed under a rigorous phylogenetic context. Currently, the number of accepted genera in Berberidaceae ranges consecutively from 13 to 19, depending on whether to define *Berberis*, *Jeffersonia*, and *Podophyllum* broadly, or to segregate these three genera further and recognize *Alloberberis*, *Mahonia*, and *Moranothamnus*, *Plagiorhegma*, and *Dysosma*, *Diphylleia*, and *Sinopodophyllum*, respectively. To resolve Berberidaceae’s taxonomic disputes, we newly assembled 23 plastomes and, together with 85 plastomes from the GenBank, completed the generic sampling of the family. With 4 problematic and 14 redundant plastome sequences excluded, robust phylogenomic relationships were reconstructed based on 93 plastomes representing all 19 genera of Berberidaceae and three outgroups. Maximum likelihood phylogenomic relationships corroborated with divergence time estimation support the recognition of three subfamilies Berberidoideae, Nandinoideae, and Podophylloideae, with tribes Berberideae and Ranzanieae, Leonticeae and Nandineae, and Podophylleae, Achlydeae, Bongardieae *tr. nov.*, Epimedieae, and Jeffersonieae *tr. nov*. in the former three subfamilies, respectively. By applying specifically stated criteria, our phylogenomic data also support the classification of 19 genera, recognizing *Alloberberis*, *Mahonia*, and *Moranothamnus*, *Plagiorhegma*, and *Diphylleia*, *Dysosma*, and *Sinopodophyllum* that are morphologically and evolutionarily distinct from *Berberis*, *Jeffersonia*, and *Podophyllum*, respectively. Comparison of plastome structures across Berberidaceae confirms inverted repeat expansion in the tribe Berberideae and reveals substantial length variation in *accD* gene caused by repeated sequences in Berberidoideae. Comparison of plastome tree with previous studies and nuclear ribosomal DNA (nrDNA) phylogeny also reveals considerable conflicts at different phylogenetic levels, suggesting that incomplete lineage sorting and/or hybridization had occurred throughout the evolutionary history of Berberidaceae and that *Alloberberis* and *Moranothamnus* could have resulted from reciprocal hybridization between *Berberis* and *Mahonia* in ancient times prior to the radiations of the latter two genera.

## Introduction

**FIGURE 1:**
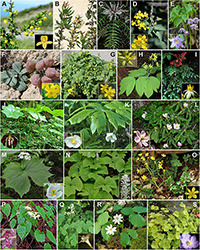
Morphological diversity in Berberidaceae. **(A)** *Berberis morrisonensis* and *B. mingetsensis* (flower). **(B)** *Moranothamnus claireae*, courtesy of Bart O’Brien. **(C)** *Mahonia oiwakensis*. **(D)** *Alloberberis fremontii* and flower photo of *A. nevinii* by Stan Shebs/CC BY-SA 3.0. **(E)** *Ranzania japonica*, courtesy of Takuro Ito, and flower photo by Qwert1234/CC BY-SA 3.0. **(F)** *Leontice incerta*, photo by Yuriy Danilevsky/CC BY-SA 3.0 and *L. leotopetalum* (flowers), photo by Averater/CC BY-SA 3.0. **(G)** *Gymnospermium altaicum*, photos by Ettrig/CC BY-SA 4.0. **(H)** *Caulophyllum robustum*, photo by Qwert1234/CC BY-SA 3.0, flower photo by Alpsdake/CC BY-SA 4.0. **(I)** *Nandina domestica*. **(J)** *Dysosma pleiantha*. **(K)** *Podophyllum peltatum*, photo by WilderAddict/CC BY-SA 4.0, flower photo by Nicholas A. Tonelli/CC BY 2.0. **(L)** *Sinopodophyllum hexandrum*, courtesy of Mu-Tan Hsieh. **(M)** *Diphylleia grayi*, courtesy of Takuro Ito, and flower photo by yamatsu/CC0 1.0. **(N)** *Achlys triphylla*, courtesy of Takuro Ito. **(O)** *Bongardia chrysogonum*, photos by Ori Fragman-Sapir/CC BY 3.0. **(P)** *Epimedium koreanum*, photo by Qwert1234/CC BY-SA 3.0 and flower photo of *E. grandiflorum* var. *thunbergianum* by Alpsdake/CC BY-SA 3.0. **(Q)** *Vancouveria hexandra*, photo by Krzysztof Ziarnek, Kenraiz/CC BY-SA 4.0, and flower photo by Walter Siegmund/CC BY-SA 3.0. **(R)** *Jeffersonia diphylla*, photo by Barnes Dr. Thomas G, U.S. Fish and Wildlife Service. **(S)** *Plagiorhegma dubium*, photo by Daderot/CC0 1.0 and flower photo by sunoochi/CC BY 2.0.

The early-diverging eudicot family Berberidaceae is composed of a morphological diverse assemblage of genera (Figure 1) long praised for their great horticultural (Ahrendt, 1961; Stearn, 2002) and medicinal values (Peng et al., 2006; Hao, 2018). Although more than 85% of the ca. 700 species of Berberidaceae (Christenhusz and Byng, 2016) are woody shrubs (Yu and Chung, 2017), at the generic level, the family is predominantly represented by mono- and oligotypic temperate herbaceous genera known for several classic examples of biogeographic disjunctions (Liu et al., 2002; Wang et al., 2007; Zhang et al., 2007; Sun et al., 2018).

In the Northern Hemisphere, Berberidaceae exhibits seven intercontinental disjunctions: the East Asian (EA) and western North American (WNA) disjunctions in *Achlys* (Fukuda, 1967) and *Mahonia* (Yu and Chung, 2017; Chen et al., 2020), the Eurasian *Epimedium* and its WNA disjunct sister genus *Vancouveria* (Stearn, 1938; Zhang et al., 2007), the EA and eastern North American (ENA) disjunctions in *Diphylleia* (Ying et al., 1984) and *Caulophyllum* (Loconte and Blackwell, 1985), and the EA monotypic genera *Sinopodophyllum* (Ying, 1979) and *Plagiorhegma* (Hutchinson, 1920) and their respective disjunct ENA sister genera *Podophyllum* and *Jeffersonia* (Wang et al., 2007). Because of great economic, ecological, and taxonomic interests, Berberidaceae has been studied extensively in seedling morphology (Terabayashi, 1985c), floral morphology (Terabayashi, 1985a; Brückner, 2000), embryology (Sastri, 1969), serology (Jensen, 1973), palynology (Zhang et al., 2017), wood anatomy (Carlquist, 1995), and chromosome cytology (Kuroki, 1970; Adhikari et al., 2014; Huang et al., 2018; Wang et al., 2020).

Historically, however, owing to the heterogeneous composition of the family that is “*held together more by a linkage of characteristics than by possession of any set of diagnostic features* (Meacham, 1980),” Berberidaceae had been variously segregated into smaller families including Nandinaceae, Leonticaceae, Podophyllaceae, and Ranzaniaceae (e.g., Janchen, 1949; Airy Shaw, 1973; Hutchinson, 1973; Wu et al., 2003; Takhtajan, 2009; Lu and Tang, 2020), and/or classified into different infrafamilial taxa including subfamilies (i.e., Berberidoideae, Epimedioideae, Leonticoideae, Nandinoideae, and Podophylloideae) and tribes (i.e., Achlydeae, Berberideae, Bongardieae, Epimedieae, Leonticeae, Podophylleae, and Ranzanieae) (Table 1). Additionally, as stated in the popular encyclopedia “Flowering Plant Families of the World” that Berberidaceae contains “12 to 16” genera (Heywood et al., 2007), generic delimitation of the family has long been disputed and thus the number of its recognized genera varies greatly (Table 1 and Supplementary Table 1). Indeed, there seems no consensus regarding whether to adopt a broadly defined *Berberis* (e.g., Sun et al., 2018; Kreuzer et al., 2019), *Jeffersonia* (e.g., Sun et al., 2016), and *Podophyllum* (e.g., Shaw, 2002; Christenhusz et al., 2018), or to recognize *Alloberberis*, *Mahonia*, and *Moranothamnus*, *Plagiorhegma*, and *Diphylleia*, *Dysosma*, and *Sinopodophyllum* as distinct genera separated from the former three genera (Supplementary Table 1). In particular, whether *Mahonia* (i.e., the compound-leaved *Berberis*) should be synonymized under a broad sense *Berberis* (*Berberis s.l.*) has been debated for more than two centuries (Fedde, 1901; Moran, 1982; Kim et al., 2004b; Adhikari et al., 2015; Yu and Chung, 2017). Please refer to Ahrendt (1961) and Yu and Chung (2017) for more details about the *Berberis* vs. *Mahonia* debates.

**TABLE 1:** Different classification systems proposed for Berberidaceae.

Present study	Janchen (1949)	Airy Shaw (1973)	Hutchinson (1973)	Meacham (1980) ^1^	Terabayashi (1985b)	Loconte and Estes (1989)
**Berberidaceae** (*N*^2^ = 19) Berberidoideae Berberideae *Alloberberis*, *Berberis*, *Mahonia*, *Moranothamnus* Ranzanieae *Ranzania* Nandinoideae Leonticeae *Caulophyllum*, *Gymnospermium*, *Leontice* Nandineae *Nandina* Podophylloideae Achlydeae *Achlys* Bongardieae *tr. nov. Bongardia* Epimedieae *Epimedium*, *Vancouveria* Jeffersonieae *tr. nov. Jeffersonia*, *Plagiorhegma* Podophylleae *Diphylleia*, *Dysosma*, *Podophyllum*, *Sinopodophyllum*	**Berberidaceae** (*N* = 15) Berberidoideae Berberideae Berberidinae *Berberis*, *Mahonia* Ranzaniinae *Ranzania* Epimedieae Epimediinae *Bongardia*, *Caulophyllum*, *Epimedium*, *Gymnospermium*, *Jeffersonia*, *Leontice*, *Plagiorhegma*, *Vancouveria* Achlyinae *Achlys* Podophylloideae Podophylleae Podophyllinae *Dysosma*, *Podophyllum* Diphylleiinae *Diphylleia* **Nandinaceae** (*N* = 1) *Nandina*	**Berberidaceae** (*N* = 4) *Berberis*, *Epimedium*, *Mahonia*, *Vancouveria* **Leonticaceae** (*N* = 4) *Bongardia*, *Caulophyllum*, *Gymnospermium*, *Leontice* **Nandinaceae** (*N* = 1) *Nandina* **Podophyllaceae** (*N* = 7) *Achlys*, *Diphylleia*, *Dysosma*, *Jeffersonia*, *Plagiorhegma*, *Podophyllum*, *Ranzania*	**Berberidaceae** (*N* = 2) *Berberis*, *Mahonia* **Nandinaceae** (*N* = 1) *Nandina* **Podophyllaceae** (*N* = 13) *Achlys*, *Bongardia*, *Caulophyllum*, *Diphylleia*, *Dysosma*, *Epimedium*, *Gymnospermium*, *Jeffersonia*, *Leontice*, *Plagiorhegma*, *Podophyllum*, *Ranzania*, *Vancouveria*	**Berberidaceae** (*N* = 15) Berberidoideae *Berberis*, *Mahonia*, *Ranzania* Podophylloideae *Diphylleia*, *Dysosma*, *Podophyllum* Epimedioideae *Achlys*, *Epimedium*, *Jeffersonia*, *Plagiorhegma*, *Vancouveria* Leonticoideae *Bongardia*, *Caulophyllum*, *Gymnospermium*, *Leontice* **Nandinaceae** (*N* = 1) *Nandina*	**Berberidaceae** (*N* = 16) Berberidoideae Berberideae *Berberis*, *Mahonia* Ranzanieae *Ranzania* Epimedieae Epimediinae *Achlys*, *Epimedium*, *Jeffersonia*, *Plagiorhegma*, *Vancouveria* Leonticinae *Bongardia*, *Caulophyllum*, *Leontice*, *Gymnospermium* Podophylleae *Diphylleia*, *Dysosma*, *Podophyllum* Nandinoideae *Nandina*	**Berberidaceae** (*N* = 17) Berberidoideae Berberideae Berberidinae *Berberis*, *Mahonia*, *Ranzania* Epimediinae *Achlys*, *Bongardia*, *Diphylleia*, *Dysosma*, *Epimedium*, *Jeffersonia*, *Plagiorhegma*, *Podophyllum*, *Sinopodophyllum*, *Vancouveria* Leonticeae *Caulophyllum*, *Leontice*, *Gymnospermium* Nandinoideae *Nandina*

**Thorne (1992)**	**Loconte (1993) and Loconte et al. (1995)**	**Takhtajan (1997) and Takhtajan (2009)**	**Thorne (2000) and Thorne and Reveal (2007)**	**Wang et al. (2009)**	**Wu et al. (2003) and Lu and Tang (2020)**

**Berberidaceae** (*N* = 16) Berberidoideae *Berberis*, *Mahonia*, *Ranzania* Leonticoideae *Caulophyllum*, *Leontice*, *Gymnospermium* Epimedioideae *Achlys*, *Bongardia*, *Dysosma*, *Diphylleia*, *Epimedium*, *Jeffersonia*, *Plagiorhegma*, *Podophyllum* (+*Sinopodophyllum*), *Vancouveria* Nandinoideae *Nandina*	**Berberidaceae** (*N* = 15) Berberidoideae Berberideae Berberidinae *Berberis*, *Mahonia*, *Ranzania* Epimediinae *Achlys*, *Bongardia*, *Dysosma*, *Epimedium*, *Jeffersonia* (+*Plagiorhegma*), *Vancouveria*, *Podophyllum* (+*Sinopodophyllum*) Leonticeae *Caulophyllum*, *Diphylleia*, *Gymnospermium*, *Leontice* Nandinoideae *Nandina*	**Berberidaceae** (*N* = 2) *Berberis*, *Mahonia* **Ranzaniaceae** (*N* = 1) *Ranzania* **Podophyllaceae** (*N* = 12) Leonticoideae *Caulophyllum*, *Gymnospermium*, *Leontice* Epimedioideae Epimedieae *Epimedium*, *Vancouveria*, *Jeffersonia*, *Plagiorhegma* Achlydeae *Achlys* Bongardieae *Bongardia* Podophylloideae *Diphylleia*, *Dysosma*, *Podophyllum* (+*Sinopodophyllum*) **Nandinaceae** (*N* = 1) *Nandina*	**Berberidaceae** (*N* = 13) Berberidoideae *Berberis* (+*Mahonia*), *Ranzania* Leonticoideae *Caulophyllum*, *Leontice*, *Gymnospermium* Podophylloideae *Achlys*, *Bongardia*, *Dysosma* (+*Diphylleia*?), *Epimedium*, *Jeffersonia* (+*Plagiorhegma*), *Podophyllum* (+*Sinopodophyllum*), *Vancouveria* Nandinoideae *Nandina*	**Berberidaceae** (*N* = 16) Berberidoideae *Berberis*, *Mahonia*, *Ranzania* Podophylloideae *Achlys*, *Diphylleia*, *Dysosma*, *Podophyllum*, *Sinopodophyllum*, *Bongardia*, *Epimedium*, *Vancouveria*, *Jeffersonia*, *Plagiorhegma* Nandinoideae *Caulophyllum*, *Gymnospermium* (+*Leontice*), *Nandina*	**Berberidaceae** (*N* = 3) Berberideae *Berberis*, *Mahonia* Ranzanieae *Ranzania* **Leonticaceae** (*N* = 3) *Caulophyllum*, *Gymnospermium*, *Leontice* **Podophyllaceae** (*N* = 10) Epimedioideae Epimedieae *Epimedium*, *Vancouveria*, *Jeffersonia*, *Plagiorhegma* Achlydeae *Achlys* Bongardieae *Bongardia* Podophylloideae *Diphylleia*, *Dysosma*, *Podophyllum*, *Sinopodophyllum* **Nandinaceae** (*N* = 1) *Nandina*

*^1^Meacham’s (1980) analysis supports the recognition of four “subfamilial taxa” without formal taxonomic treatment; the four subfamilies presented here are added based on taxonomic priority. ^2^N, the number of genera.*

To resolve Berberidaceae’s taxonomic controversies, early molecular studies using nuclear glyceraldehyde-3-phosphate dehydrogenase gene (Adachi et al., 1995) and chloroplast *rbcL* gene and restriction site (Kim and Jansen, 1995) both showed that *Nandina* should be included within the family. Subsequent molecular phylogenetic studies (Kim and Jansen, 1996; Kim et al., 2004a; Wang et al., 2007) revealed three clades within Berberidaceae, resulting in the circumscription of three subfamilies corresponding to three chromosome groups (Wang et al., 2009): Berberidoideae (*x* = 7), Podophylloideae (*x* = 6), and Nandinoideae (*x* = 8 and *x* = 10). Except for Lu and Tang (2020), Wang et al.’s (2009) subfamilial classification of Berberidaceae has been widely followed (Table 1). Subsequent historical biogeographic analyses based on molecular phylogenetic data also indicate that the Bering Land Bridge had functioned as a crucial pathway for the intercontinental disjunctions (Wen et al., 2010). Based on the internal transcribed spacer (ITS), Kim et al. (2004b) showed that *Mahonia* is paraphyletic, with *Mahonia* sect. *Horridae* sister to the simple-leaved *Berberis* (i.e., *Berberis s.s.*). More recently, based on the combined analysis of ITS and chloroplast *ndhF* gene sequences, Adhikari et al. (2015) further showed that Sect. *Horridae* is polyphyletic, together with Kim et al. (2004b) arguing for a broadly circumscribed *Berberis* (i.e., *Berberis s.l.*) that includes the compound-leaved *Mahonia*.

However, both Kim et al. (2004b) and Adhikari et al. (2015) suffered from issues including inadequate taxon sampling, problematic outgroup rooting, inclusion of poor-quality DNA sequences from GenBank, and taxon misidentification, undermining their taxonomic conclusion (Yu and Chung, 2017). To resolve the *Mahonia* vs. *Berberis* debate that has been lasting for more than two centuries, Yu and Chung (2017) expanded and verified taxon sampling of *Mahonia* and included *Berberis claireae*, a unique spineless Baja California endemic species with unifoliolate to 7-foliolate compound leaves (Moran, 1982) that had never been sampled previously. Based on ITS and four cpDNA markers, Yu and Chung’s (2017) phylogenetic analyses of *Berberis s.l.* revealed four strongly supported clades, *Berberis s.s.*, *B. claireae*, core *Mahonia*, and *Mahonia* sect. *Horridae*. Because these four clades are ecologically and morphologically distinct and evolutionarily comparable to other genera of Berberidaceae, Yu and Chung (2017) proposed a new classification that recognizes these four clades as genera: *Alloberberis* (≡ *Mahonia* sect. *Horridae*), *Berberis* (≡ *Berberis s.s.*), *Mahonia* (≡ core *Mahonia*), and, *Moranothamnus* (≡ *B. claireae*), “reloading” the two-century long “*Mahonia* vs. *Berberis*” debate (see cover of the journal *Taxon* 66(6); doi.org/10.1002/tax.666001).

However, debates on generic concepts of Berberidaceae are not restricted to *Mahonia* vs. *Berberis*. Neither do controversies end with phylogenetic and phylogenomic data. In both Kim et al. (2004a) and Wang et al. (2007), Berberidaceae were regarded as having 17 genera; however, in the first molecular-based formal infrafamilial classification of Berberidaceae, Wang et al. (2009) sampled “*all 16 genera of Berberidaceae*,” neglecting *Leontice* L. that had never previously been synonymized (Table 1). In a recent phylogenomic study using plastome sequences, Sun et al. (2018) recognized 18 genera in Berberidaceae, accepting Yu and Chung’s (2017) new genera *Alloberberis* and *Moranothamnus* and yet subsuming *Plagiorhegma* under *Jeffersonia* (Table 2). However, in a subsequent study aiming to develop clade-specific DNA barcodes of *Berberis* using plastome sequences, sampled *Alloberberis nevinii*, *Mahonia nervosa*, and *M. polyodonta* were all treated as *Berberis s.l.* (Kreuzer et al., 2019). The flux of Berberidaceae’s generic delimitation is also manifested across major biodiversity databases and online resources (Supplementary Table 1). Nevertheless, Yu and Chung’s (2017) classification has been taken by taxonomic (Colin et al., 2021), floristic (Galasso et al., 2018), paleobotanical (Doweld, 2018), and biogeographic (Chen et al., 2020) studies.

**TABLE 2:** Summary of the plastome and nrDNA assembly data.

		Plastome								nrDNA			
Species	# all reads^1^	NCBI accession	Length (bp)	LSC (bp)	SSC (bp)	IR (bp)	%GC	Av. cov. (×)	Cov. SD	NCBI accession	Length (bp)	Av. cov. (×)	Cov. SD
*Alloberberis fremontii*	8,924,456	MT335778	165,871	73,262	18,779	36,915	38.1	496.2	141.8	MW545966	7300	1454	1096.3
*A. higginsiae*	10,224,582	MT335779	165,883	73,279	18,788	36,908	38.1	902.0	269.6	MW545967	7300	1823.3	1367
*A. trifoliolata*	10,646,702	MT335780	164,553	72,349	18,738	36,733	38.1	101.5	31.1	MW545968	6906	1893.0	2156.7
*Berberis dictyophylla*	9,547,576	MT335782	166,036	73,449	18,611	36,988	38.1	57.0	16.3	MW545974	7198	987.0	258.3
*B. hayatana*	10,975,726	MT335783	168,208	73,245	16,277	39,343	38.0	333.7	66.5	MW545975	7220	1171.9	304.7
*B. kawakamii*	9,066,338	MT335784	167,658	73,294	16,194	39,085	38.1	212.7	45.7	MW545976	7221	933.9	243.6
*B. morrisonensis*	11,053,602	MT335785	166,145	73,490	18,623	37,016	38.1	331.2	74.2	MW545979	7198	1476.5	280.6
*B. nantoensis*	9,136,400	MT335806	167,898	73,296	16,270	39,166	38.0	267.7	52.5	MW545980	7220	944.5	179
*B. pruinosa*	9,574,826	MT335786	165,455	73,348	18,573	36,767	38.1	95.4	23.4	MW545982	7260	1217.0	318.5
*B. saxicola*	9,458,418	MT335787	166,172	73,606	18,692	36,937	38.1	128.2	34.2	MW545984	6843	940.1	268.4
*B. vulgaris*	10,088,158	MT335788	166,150	73,460	18,660	37,015	38.0	324.4	71.3	MW545987	7192	2063.8	561.9
*Mahonia aquifolium*	9,557,402	MT335789	165,517	73,149	18,758	36,805	38.1	546.9	173.8	MW545988	7110	1372.8	597.8
*M. chochoco*	11,204,800	MT335790	165,367	73,301	18,682	36,692	38.1	251.4	100.6	MW545989	7322	750.3	702
*M. dictyota*	10,890,484	MT335791	165,495	73,065	18,824	36,803	38.1	172.5	45.2	MW545990	7110	955.8	240.2
*M. fortunei*	10,301,560	MT335792	165,654	73,669	18,623	36,681	38.0	133.2	30.5	MW545991	7165	866.4	278.8
*M. harrisoniana*	10,575,142	MT335793	165,367	73,095	18,822	36,725	38.1	967.1	263.1	MW545992	7110	920.5	367.6
*M. japonica*	9,972,444	MT335794	164,827	73,253	18,634	36,470	38.2	484.4	97.6	MW545993	7313	2547.7	849.7
*M. lanceolata*	9,623,936	MT335795	165,796	72,886	18,744	37,083	38.0	297.7	66.3	MW545994	7168	1294.6	494.2
*M. nervosa*	11,134,750	MT335796	165,707	73,128	18,825	36,877	38.1	119.4	30.5	MW545995	7346	1354.6	500.5
*M. oiwakensis*	7,600,014	MT335797	165,021	73,260	18,649	36,556	38.1	609.3	115.9	MW545996	7324	853.7	329.3
*M. pallida*	11,120,770	MT335798	165,707	72,782	18,717	37,104	38.0	298.5	67.8	MW545997	7202	1373.4	505.4
*M. tikushiensis*	8,869,818	MT335799	164,876	73,273	18,713	36,445	38.1	226.5	11.3	MW545998	7313	1062.6	378
*Moranothamnus claireae*	10,834,754	MT335800	165,706	73,324	18,932	36,725	38.1	95.9	25.4	MW545999	7232	1176.0	686.9

*LSC, large single copy; SSC, small single copy; IR, inverted repeat; %GC, GC content percentage; av. cov., average coverage; #, the number of; cp, chloroplast; SD, standard deviation.*

*^1^Number of all the trimmed reads of the sample.*

The disparity on generic concepts across different studies and online resources (Supplementary Table 1) illustrates the lack of consensus on precise and objective criteria for generic delimitation (Humphreys and Linder, 2009) in Berberidaceae. Indeed, most of the abovementioned studies and online resources did not specify references or explicitly state reasons for their adoption of a particular generic treatment. To achieve an objective generic delimitation of *Berberis s.l.*, Yu and Chung (2017) followed strictly five criteria advocated by Backlund and Bremer (1998), Linder et al. (2010), and Heenan and Smissen (2013) to delimit *Berberis s.l.*: (1) prioritizing primary (i.e., family, genus, and species) over secondary ranks (i.e., subgenus, section, etc.), (2) maximizing phylogenetic information and reducing redundancy in a classification, (3) recognizing evolutionarily equivalent (i.e., clade age, phylogenetic distance, and morphology) groups as the same rank, (4) delimiting genus that is morphologically, ecologically, and geographically homogenous, and (5) taking into account the full taxonomic history of the group and minimizing name changes to maintain nomenclatural stability. However, such objective generic delimitation has not been applied to other genera of Berberidaceae.

In recent years, rapid advances in high-throughput sequencing technology have made plastome sequences accessible for resolving recalcitrant phylogenetic relationships not attainable previously using Sanger sequences (Wicke and Schneeweiss, 2015; Tonti-Filippini et al., 2017; Gitzendanner et al., 2018). Several phylogenomic studies of Berberidaceae have been conducted using whole plastome sequences (Zhang et al., 2016; Sun et al., 2018; Ye et al., 2018; Kreuzer et al., 2019); however, no phylogenomic studies have yet sampled all 19 genera and covered adequate infrageneric diversity needed to resolve the taxonomic controversies. In this study, we report 23 newly assembled plastome sequences that complete the generic sampling of Berberidaceae. By implementing explicit criteria of generic delimitation, an infrafamilial classification representing monophyletic subdivisions of Berberidaceae is proposed, aiming to settle the taxonomic controversies and debates that have been fraught for centuries.

## Materials and Methods

### Classification Adopted

For clarity, the classification of 19 genera in Berberidaceae (Table 1) that recognizes *Alloberberis*, *Berberis*, *Mahonia*, and *Moranothamnus* (Yu and Chung, 2017), *Jeffersonia* and *Plagiorhegma* (Hutchinson, 1920), and *Diphylleia*, *Dysosma*, *Podophyllum*, and *Sinopodophyllum* (Wang et al., 2009) as opposed to the broadly defined *Berberis s.l.*, *Jeffersonia s.l.*, and *Podophyllum s.l.*, respectively, is followed in all subsequent discussion unless otherwise stated.

### Taxon Sampling

A total of 85 plastomes representing 60 species and two additional varieties in 17 genera of Berberidaceae available (accessed 25 March 2021) on GenBank were downloaded (Supplementary Table 2). To complete generic (*N* = 19) and infrageneric sampling of Berberidaceae, 23 species of Berberideae, including 3 species of *Alloberberis*, the monotypic *Moranothamnus*, 8 species of *Berberis* (7 species of Group Septentrionales and 1 species of Group Australes), and 11 species of *Mahonia* (5 species of Group Orientales and 6 of Group Occidentales), were sampled (Supplementary Table 2) for plastome assembly. Although we only sampled 11 species and two additional varieties of the ca. 500 species of *Berberis* and 11 of the ca. 100 species of *Mahonia*, our sampling is geographically and phylogenetically sufficient (Yu and Chung, 2017; Yu, 2018) to address issues of generic circumscription in Berberidaceae. Based on recent studies (e.g., Lane et al., 2018), plastomes of *Ranunculus macrantha* (Ranunculaceae), *Stephania japonica* (Menispermaceae), and *Akebia quinata* (Lardizabalaceae) were also downloaded from GenBank as outgroups (Supplementary Table 2).

### DNA Extraction and Next-Generation Sequencing

CTAB method (Doyle and Doyle, 1987) was used to extract total genomic DNA from silica-dried and herbarium leaf materials. The DNA concentration was quantified by Qubit 3.0 Fluorometer (Thermo Fisher Scientific, Waltham, MA, United States). The DNAs were sent to the Genomic Core Lab of Institute of Molecular Biology, Academia Sinica for library preparation using KAPA LTP Library Preparation Kits (KAPA Biosystems, Wilmington, MA, United States), and for whole genome shotgun (WGS) sequencing using Illumina NextSeq 500 (Illumina Inc., San Diego, CA, United States) with pair-end mode, read length = 150 bp, and insert size = ca. 300 bp.

### Plastome Assembly and Annotation

The quality of raw reads was assessed by FastQC v.0.11.9 (Andrews, 2010). Reads were trimmed using Trimmomatic v.0.39 (Bolger et al., 2014) with the setting “LEADING:25 TRAILING:25 SLIDINGWINDOW:4:20 CROP:149 MINLEN:100.” The *de novo* assembly of the plastome was performed by GetOrganelle v.1.7.5 (Jin et al., 2020) with the setting of “-R 10 -t 3 -w 0.8 -k 37,55,65,85,105,127,131 -F embplant_pt –reduce-reads-for-coverage inf,” using *Berberis amurensis* (GenBank accession: KM057374) as a reference for assembly. The resulting sequences generated by GetOrganelle were imported into Geneious Prime (Biomatters Ltd., Auckland, New Zealand) (Kearse et al., 2012) for validation and/or final assembly completion. For samples not assembled into a complete plastome using GetOrganelle, the “Map to Reference” function with “High Sensitivity” and default setting of Geneious was implemented to generate the draft genome, using the consensus of the mapping file to temporarily fill the “unassembled regions.” The “unassembled regions” were corrected by mapping the trimmed reads to the draft genome using the “Map to Reference” function with “Medium-Low Sensitivity” and default setting in order to complete the assembly. All complete plastome sequences were further verified by read mapping.

Newly assembled plastomes were annotated by transferring the annotations of published Berberidaceae plastomes to the newly sequenced ones under the alignment generated by MAFFT v.7.388 (Katoh and Standley, 2013) launched in Geneious. The presence of start and stop codons of each protein-coding gene was checked and adjusted manually. Genes with any premature stop codon that might interrupt translations from half of the original reading frame were annotated as pseudogenes. The correct length and identity of tRNA genes were further confirmed using the web server tRNAscan-SE 2.0 (Lowe and Chan, 2016). The boundaries of IRs were annotated by GeSeq (Tillich et al., 2017) and manually checked with self-dot plots under Geneious. Plastome maps were drawn using OGDRAW (Greiner et al., 2019).

### Plastome Phylogenetic Analyses

Our initial matrix comprised 111 plastomes, including 108 of Berberidaceae (81 species and 2 additional varieties in 19 genera) and 3 outgroups (Supplementary Table 2). The sequence MG593045 (*Dysosma delavayi*) was excluded because high sequence variation was detected between its two inverted repeats (IRs). Of the remaining 80 species of Berberidaceae, 18 species were represented by multiple sequences. To lessen computational loading, we conducted a preliminary maximum likelihood (ML) analysis of the 110 sequences using IQ-TREE v.1.6.12 (Nguyen et al., 2015). Based on the preliminary ML tree (Supplementary Figure 1), 14 redundant and 3 problematic sequences were further excluded (see section “Results”), leaving a total of 93 plastomes representing 80 species and 2 additional varieties in all 19 genera of Berberidaceae and 3 outgroups for subsequent analyses.

Prior to phylogenetic analyses, IRB was removed. To accommodate substitution rate heterogeneity across plastomes, sequences were partitioned by the four gene categories [i.e., coding sequences (CDSs) of protein-coding genes, introns, RNA (tRNA and rRNA) genes, and intergenic spacers (IGSs)] as well as codon position of CDS. Each category was extracted, concatenated, and aligned individually by MAFFT using Geneious. For CDS, after excluding pseudogenes and partially duplicated genes, the remaining 76 genes (Supplementary Table 3) were concatenated and aligned using the “Translation Align” function based on bacterial genetic codes implemented by MAFFT under Geneious, with manual adjustments. The final concatenated alignment contains six partitions (i.e., plastid partition scheme): CDS1, CDS2, CDS3, introns, RNA genes, and IGS. Sites with more than 97% gaps were excluded using “Mask Alignment” function in Geneious. The number and proportion of parsimony informative sites of the concatenated plastome alignment were calculated by AMAS (Borowiec, 2016).

IQ-TREE was used with the “-m MFP+MERGE -bb 5000” option to conduct the following analyses: (1) searching for the best-fit partition scheme, (2) determining the best-fit nucleotide model for each partition by ModelFinder (Kalyaanamoorthy et al., 2017), and (3) reconstructing phylogenies based on ML method with 5000 replicates using ultrafast bootstrap approximation approach (Minh et al., 2013). The final tree with ultrafast bootstrap support (UFBS) values was visualized using FigTree v.1.4.2.^1^

### Nuclear Ribosomal DNA Assembly and Analysis

Nuclear ribosomal DNA (nrDNA) sequences, spanning across partial external transcribed spacer (ETS), 18S rRNA gene, ITS 1, 5.8S rRNA gene, ITS2, 26S rRNA gene, and partial non-transcribed spacer (NTS) were assembled from raw reads of the 23 newly generated WGS sequencing using GetOrganelle with the setting of “-R 15 -t 10 -w 0.7 -k 37,69,85,115,127,131,135,139 -F embplant_nr –reduce-reads-for-coverage inf.” Additionally, nrDNA were also assembled for *B. amurensis*, *B. koreana*, *B. weiningensis*, *Bongardia chrysogonum*, and *Podophyllum peltatum* from WGS sequencing reads downloaded from NCBI Sequence Read Archive (SRA) using NCBI SRA Toolkit v.2.1.11. The nrDNA of these samples were assembled by executing GetOrganelle with customized settings (Supplementary Table 7). All nrDNA were verified by read mapping with the same procedure as verifying plastome sequences.

The 28 nrDNA sequences were aligned and partitioned (i.e., partial ETS, 18S, ITS1, 5.8S, ITS2, 26S, and partial NTS) by MAFFT implemented in Geneious. We employed IQ-TREE with “-m MFP+MERGE -bb 5000” options to conduct the same analyses as the plastome dataset. Concurrently, a plastome tree including 28 species sampled for the nrDNA was generated by IQ-TREE using the same partition scheme and analytical settings of the 93-plastome dataset.

### Divergence Times Estimation

For divergence times estimation, we kept only one sequence for each species to further reduce the computational time. As a result, the matrix including 83 plastome sequences of 80 species in 19 genera of Berberidaceae and 3 outgroups (Supplementary Table 2) was analyzed using BEAST v.2.6.0 (Bouckaert et al., 2019) on CIPRES Science Gateway v.3 (Miller et al., 2011). Parameters and priors of the input xml file were set *via* BEAUti launched in the software package of BEAST v.2.6.0. With IRB excluded, the analysis was performed with the plastid partition scheme, and the prior of site models were set according to the best-fit nucleotide models and partition scheme determined by ModelFinder in IQ-TREE with the options “-m TESTMERGEONLY -mset mrbayes.” To accommodate rate heterogeneity across different Berberidaceae lineages (Yu and Chung, 2017; Sun et al., 2018), we used relaxed clock log normal as the prior of the clock model. The tree prior was set as a Yule model, and the remaining parameters followed default settings except for specifying three fossil calibration points to constrain the ages of three nodes. In Yu and Chung (2017), the age of the fossil *Leefructus mirus* (Sun et al., 2011; Wang et al., 2016) at 124.4 million years ago (Ma) was taken as the crown age of the Berberidaceae + Ranunculaceae clade. However, because of concern over the authenticity of the fossil of *L. mirus* (Zhou, 2014), three alternative fossils were used instead. First, the fossil of *Prototinomiscium vangerowii* dated back to the Turonian at ca. 91 Ma was assigned as the stem age of Menispermaceae (Anderson et al., 2005; Wang et al., 2012) with a lognormal distribution (mean = 92 in real space, SD = 0.06). Second, the fossil of *Mahonia simplex* from the Oligocene dated back to ca. 28.45 Ma (Huang et al., 2016) was designated as the crown age of *Mahonia* with the lognormal distribution (mean = 28.45 in real space, sigma = 0.1). Third, the fossil of *Alloberberis obliqua* from the Oligocene at ca. 35.55 Ma (MacGinitie, 1953; Doweld, 2018) was chosen as the crown age of *Alloberberis* with a lognormal distribution (mean = 35.55 in real space, SD = 0.05). We conducted two independent runs of Markov Chain Monte Carlo (MCMC), one with 400 million generations of MCMC and the other with 200 million generations. Both runs were sampled every 1000 steps for log files and every 50,000 steps for tree files. To evaluate the convergence of each parameter, the log file of each run was summarized and visualized by Tracer v.1.7.1 (Rambaut et al., 2018). The tree files were then combined by LogCombiner v.2.6.2 (launched in BEAST v.2.6.0) with the first 100 million trees discarded as burn-in for each run. Finally, we used TreeAnnotator v.2.6.0 (launched in the software package of BEAST v.2.6.0) to summarize the combined tree file into a maximum clade credibility tree with 95% highest posterior density (HPD) interval of age of each node calculated by mean heights, and visualized the tree using FigTree.

## Results

### Plastome Features of Berberidoideae

**FIGURE 2:**
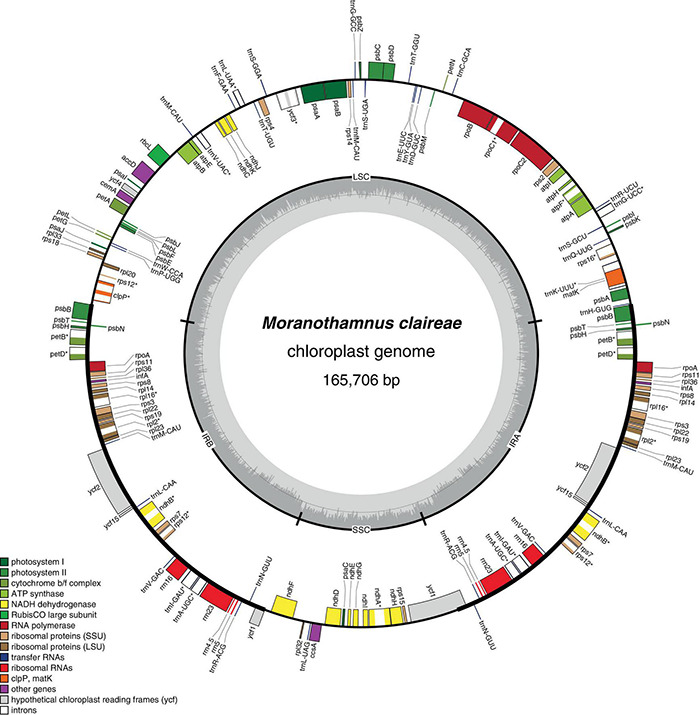
Chloroplast genome map of *Moranothamnus claireae*. The genes drawn on the inner side of the outer circle are transcribed clockwise, and those on the outer side are transcribed counterclockwise. IRs are shown in bold lines in the outer circle. The darker gray areas of the inner circle indicate GC contents across the genome with lighter gray areas indicating AT contents. Genes belonging to different functional groups were shown in different color as in the legend.

All newly generated plastomes of Berberideae were assembled into circular molecules with sizes ranging from 164,553 (*Alloberberis trifoliolata*) to 168,208 bp (*Berberis hayatana*). The average coverages of the newly assembled plastomes ranged from 64× (*B. dictyophylla*) to 1127.8× (*Mahonia harrisoniana*) (Table 2). The GC contents vary only slightly (38.0–38.2%), and the genome structures are found to represent the typical quadripartite configuration (Figure 2 and Supplementary Figures 2–5), consisting of a large single copy (LSC) ranging from 72,349 (*A. trifoliolata*) to 73,669 bp (*Mahonia fortunei*), a small single copy (SSC) ranging from 16,194 (*B. kawakamii*) to 18,932 bp (*Moranothamnus claireae*), and two IRs ranging from 36,445 (*M. tikushiensis*) to 39,343 bp (*B. hayatana*) (Table 2). Referring to early-diverging eudicots (Sun et al., 2016), both gene orders (Figure 2 and Supplementary Figures 2–5) and gene contents (Supplementary Table 3) of the 23 newly assembled plastomes are consistent with the published plastome of *Mahonia bealei* (Ma et al., 2013), which has experienced significant IR expansions at IRB/LSC boundary from *rps19* into the spacer between *clpP* and *psbB* (Supplementary Table 2 and Supplementary Figure 6). In addition to Berberideae, IR expansion was also detected in MG234280 of *Ranzania* (Wang et al., 2018) and *Epimedium ecalcaratum* (MN939634). On the other hand, IR contraction was found in MN371716 of *Epimedium brevicornu* (Zheng et al., 2019). However, both IR expansion and contraction are not previously known in *Epimedium*. Comparison of IR/SC boundaries across Berberidaceae is shown in Supplementary Figure 6. Additionally, as noted in Ma et al. (2013), *rpo*A gene is lost in all our newly sequenced plastomes of Berberideae. However, while Ma et al. (2013) reported that *ndhK* had degenerated into a pseudogene in *M. bealei*, *ndhK* gene does not contain any internal stop codon in all our newly assembled plastomes.

Together with all newly assembled plastomes, we also noticed a substantial length variation in *accD* genes in Berberidaceae (Supplementary Figure 7), especially in Berberidoideae (Supplementary Figure 8). All sampled plastomes of *Alloberberis* and *Mahonia* share a 216-bp deletion close to the 3′ end of the reading frame, with two additional deletions of 120 and 30 bp unique to the former genus (Supplementary Figure 8). However, the greatest sequence variation of *accD* locates in the central part of the gene. Visualizing the translation alignment revealed that the length variation in *accD* is featured by repeats composed of five amino acid sequences. In Berberidoideae, a total of 33 types of the amino acid repeats translated from 37 types of 15-bp DNA sequences were identified (Supplementary Table 4). The total number of these repeats in each species varies from 6 in *B. dictyophylla* to 27 in *Berberis aristata* (MN746308) and *B. saxicola*. Of the 33 amino acid repeats, R19 (120 copies) and R22 (87 copies) are the two most numerous copies, found in almost all plastomes of Berberideae (Supplementary Table 5 and Supplementary Figure 8). Some repeats were detected in certain groups and thus appear to be clade specific. For example, R21 and R31 occur exclusively in Asian *Mahonia* clade (Group Orientales) except for *M. nervosa*, R10 is unique to *Mahonia*, and R8, R20, and R23 were found only in *Alloberberis* (Supplementary Table 5 and Supplementary Figure 8).

### Plastid Phylogenomic Analyses

Our preliminary ML analyses of the 110 plastome dataset indicated that, of the 18 species represented by multiple sequences, 10 species (*B. amurensis*, *M. fortunei*, *Ranzania japonica*, *Dysosma pleiantha*, *Diphylleia sinensis*, *Sinopodophyllum hexandrum*, *E. brevicornu*, *E. tianmenshanensis*, *E. wushanense*, and *Plagiorhegma dubium*) were recovered as monophyletic groups and two species (*Achlys triphylla* and *E. pseudowushanese*) were paraphyletic (Supplementary Figure 1). Of the two plastome sequences of *A. triphylla*, MG461315 was deleted for its poor sequence quality (Ye et al., 2018). For the two plastome sequences of *R. japonica*, MH423072 (Sun et al., 2018) was selected because MG234280 (Wang et al., 2018) contains expanded IRs (Supplementary Figure 6) that was not reported by early chloroplast restriction site mapping study (Kim and Jansen, 1994). For the remaining eight monophyletic and one paraphyletic species, one plastome sequence was randomly selected for each species for subsequent analyses (Supplementary Figure 1). Seven species (*B. aristata*, *Mahonia oiwakensis*, *M. bealei*, *Dysosma versipellis*, *P. peltatum*, *Epimedium mikinorii*, and *E. sagittatum*) were shown to be polyphyletic (Supplementary Figure 1) and all their sequences were retained except for MG593052 (*P. peltatum*) that shares 99.19% of “% Identity” with *S. hexandrum* (KT445939) and yet only 87.2% with its conspecific sequence.

**FIGURE 3:**
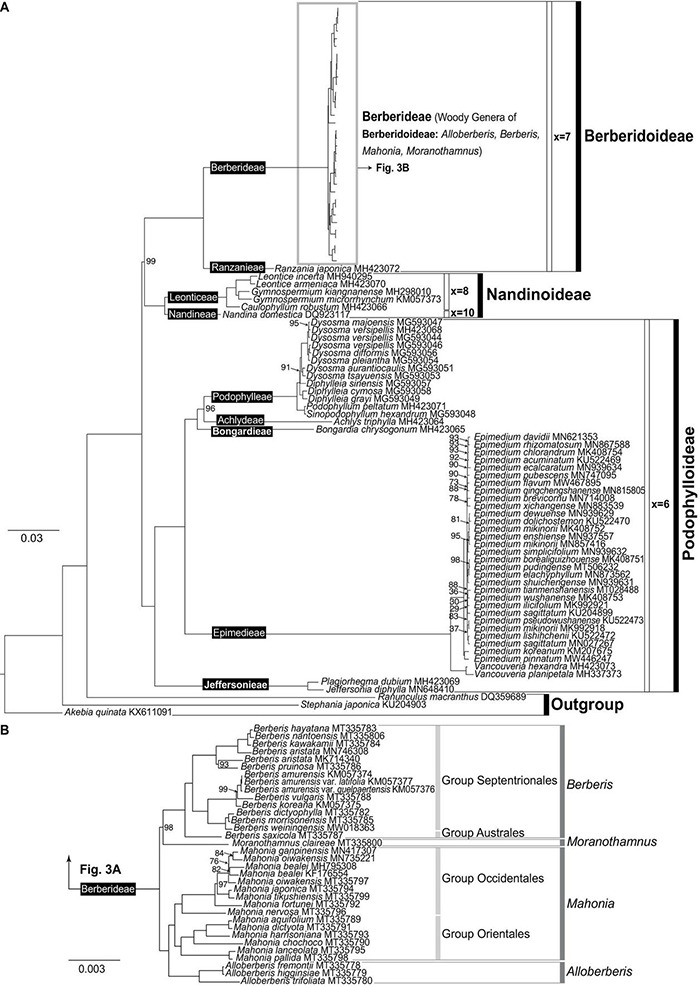
Best-scoring maximum likelihood tree based on the whole plastome sequences reconstructed under IQ-TREE. All branches are fully supported (UFBS: 100) except for those marked by exact numbers (UFBS <100). **(A)** ML phylogram of Berberidaceae labeled with infrafamilial classification proposed in current study; **(B)** phylogenetic relationships of Berberideae with labels following Yu and Chung (2017).

With IRB excluded and partitions concatenated, alignment of the remaining 93 plastomes is 153,461 bp in length, and contains 36,726 parsimony informative sites (23.93% of the alignment). The substitution models for each partition determined by ModelFinder are listed in Supplementary Table 6. Rooted by *A. quinata*, ML phylogeny shows that Ranunculaceae (*Ranunculus macranthus*) is sister to Berberidaceae (Figure 3A) with a strong support (UFBS: 100). Within Berberidaceae, three clades corresponding to the three subfamilies Berberidoideae, Nandinoideae, and Podophylloideae were recovered with full support (UFBS: 100).

Within Nandinoideae, *Nandina* (i.e., Nandineae; *x* = 10) is sister to the clade of *Leontice* + *Gymnospermium* + *Caulophyllum* (i.e., Leonticeae; *x* = 8). Within Podophylloideae, five long-branched clades (Figure 3A) corresponding to clade *Jeffersonia* + *Plagiorhegma* (i.e., Jeffersonieae *tri. nov.*), clade *Epimedium* + *Vancouveria* (i.e., Epimedieae), *Bongardia* (i.e., Bongardieae *tr. nov.*), *Achlys* (i.e., Achlydeae), and clade *Dysosma* + *Diphylleia* + *Podophyllum* + *Sinopodophyllum* (i.e., Podophylleae) were recovered, with each successive sister to the remaining clades within the subfamily (Figure 3A). Within Epimedieae, the monophyletic WNA *Vancouveria* is sister to the monophyletic Eurasian *Epimedium*. Within *Epimedium*, interspecific relationships in general are poorly supported and different relationships have been recovered between the 110-plastome (Supplementary Figure 1) and 93-plastome datasets (Figure 3); however, in both datasets, *E. pinnatum* (Subgenus *Rhizophyllum*) and *E. koreanum* (Sect. *Macroceras*) form a strongly supported clade sister to the clade of Sect. *Diphyllon* (UFBS: 100). Within Sect. *Diphyllon*, two moderately to strongly supported clades A and B each characterized by slightly different IRB/LSC boundaries were recovered (Supplementary Figure 9). Within Podophylleae, *Podophyllum* and *Sinopodophyllum* form a clade sister to *Dysosma* + *Diphylleia*, though *Diphylleia* is paraphyletic with *D. sinensis* sister to *Dysosma* (Figure 3A).

Within Berberidoideae (Figure 3A), our ML analysis also reveals that *R. japonica* (i.e., Ranzanieae) is sister to Berberideae that is composed of four clades corresponding to *Alloberberis*, *Berberis*, *Mahonia*, and *Moranothamnus* with full supports, confirming Yu and Chung’s (2017) classification. However, while *Alloberberis* was resolved as the sister clade of *Berberis* + *Mahonia* + *Moranothamnus* in Yu and Chung (2017), the genus was placed as the sister group of *Mahonia* with full support in current analysis. Although our sampling of *Berberis* is too limited to test the infrageneric classification of *Berberis* and *Mahonia* (Ahrendt, 1961), the monophyly of Group Septentrionales sister to Group Australes is strongly supported (Figure 3B). Within *Mahonia*, the monophyly of the New World Group Occidentales and the predominant Old World Group Orientales are also both fully supported (Figure 3B).

### Nuclear Ribosomal DNA and Analyses

Table 2 and Supplementary Table 7 summarizes details of the nrDNA assembly. The average coverage of each species, which was calculated by read mapping, ranges from 876.4× in *M. chochoco* to 3325.9× in *M. japonica* (Table 2). The final matrix consists of 7530 aligned base pairs with 855 parsimony informative sites (11.35% of the alignment). The best-fit substitution model for each partition under the best-fit partition scheme was determined by ModelFinder (Supplementary Table 7).

**FIGURE 4:**
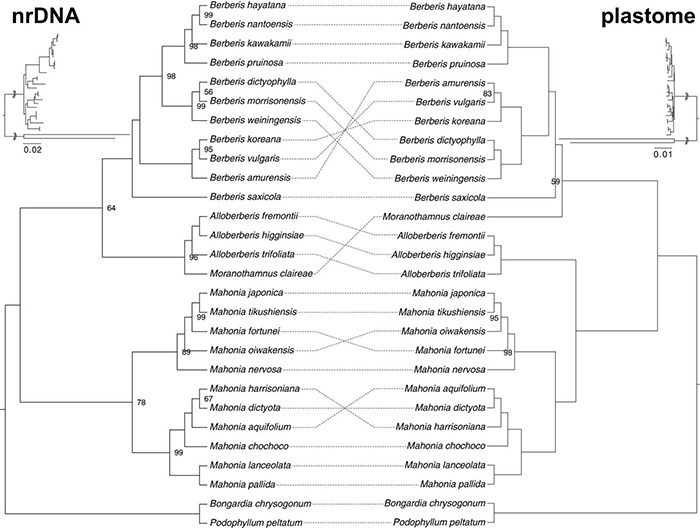
Tanglegram of nrDNA and plastome phylogenies. Nodes without number indicate UFBS = 100.

Rooted by *Bongardia* and *Podophyllum*, ML analysis of nrDNA using IQ-TREE supports the monophyly of *Alloberberis*, *Berberis*, and *Mahonia* (Figure 4), though the support for the monophyly of *Mahonia* is low (UFBS: 78). Within Berberideae, *Mahonia* is sister to the clade composed of *Berberis* + *Alloberberis* + *Moranothamnus*, with the clade *Alloberberis* + *Moranothamnus* sister to *Berberis* with low support (UFBS: 64). As shown in Figure 4, relationships in nrDNA tree among the four genera of Berberideae are in conflict with the plastome tree in which *Alloberberis* and *Moranothamnus* are placed sister to *Mahonia* and *Berberis*, respectively, though the support for the latter sister relationship is low (UFBS: 59).

### Divergence Time Estimation

**FIGURE 5:**
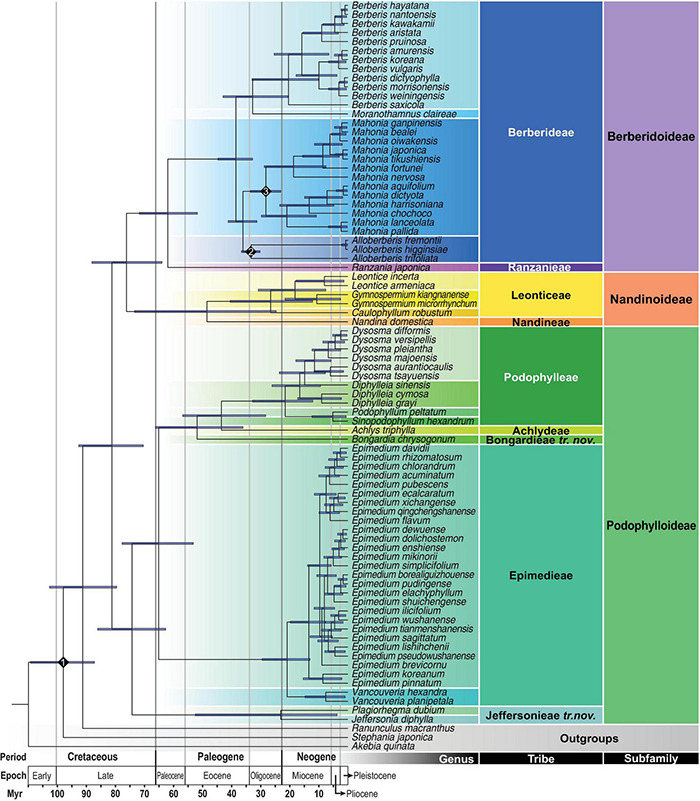
Maximum clade credibility chronogram estimated by BEAST2. The 95% highest posterior density (HPD) date ranges are shown by the node bars. Numbered diamonds are three calibration points.

The best-fit substitution model and partition scheme as the site model prior for BEAST2 analyses evaluated by ModelFinder were summarized in Supplementary Table 7. Using BEAST2, the stem and crown ages of Berberidaceae were estimated to be 91.69 Ma (95% HPD: 103.18–79.93 Ma) and 81.57 Ma (95% HPD: 93.08–70.71 Ma), respectively, falling within the Late Cretaceous (Table 3 and Figure 5). Within Podophylloideae, tribe Jeffersonieae *tr. nov*. diversified from the rest of Podophylloideae at ca. 74.62 Ma (95% HPD: 86.50–62.97 Ma), with the split between *Jeffersonia* and *Plagiorhegma* at ca. 23.15 Ma (95% HPD: 52.72–3.65 Ma). Within the clade of the remaining Podophylloideae, Epimedieae separated from Bongardieae + Achlydeae + Podophylleae at ca. 65.41 Ma (95% HPD: 78.04–53.40 Ma), while Bongardieae split from Achlydeae + Podophylleae at ca. 52.09 Ma (95% HPD: 66.53–36.22 Ma). The split between Achlydeae and Podophylleae was estimated at ca. 43.68 Ma (95% HPD: 57.10–28.36 Ma). The split of Berberidoideae from Nandinoideae was estimated to have occurred at ca. 76.54 Ma (95% HPD: 88.47–64.26 Ma). Within Nandinoideae, Nandineae diverged from the Leonticeae at ca. 48.70 Ma (95% HPD: 73.76–24.75 Ma). Within Berberidoideae, the crown age of Berberidoideae was estimated at ca. 62.24 Ma (95% HPD: 72.09–52.20 Ma). The crown ages of the clades *Alloberberis* + *Mahonia* and *Berberis* + *Moranothamnus* were estimated at ca. 36.23 Ma (95% HPD: 41.36–31.41 Ma) and ca. 32.89 Ma (95% HPD: 43.13–20.59 Ma), respectively. The crown ages of *Alloberberis*, *Berberis*, and *Mahonia* were estimated to be ca. 33.42 Ma (95% HPD: 36.77–31.32 Ma), 20.47 Ma (95% HPD: 33.09–10.08 Ma), and 28.30 Ma (95% HPD: 33.67–23.14 Ma), respectively.

**TABLE 3:** Summary of divergence times estimated for genera, tribes, and subfamilies of Berberidaceae by BEAST2.

	Crown age (myr)	Stem age (myr)
Berberidaceae	81.57 (93.08–70.71)	91.69 (103.18–79.93)
Berberidoideae	62.24 (72.09–52.00)	76.54 (88.47–64.26)
Berberideae	38.67 (44.93–32.89)	62.24 (72.09–52.00)
*Berberis + Moranothamnus*	32.89 (43.13–20.59)	38.67 (44.93–32.89)
*Moranothamnus*	N/A	32.89 (43.13–20.59)
*Berberis*	20.47 (33.09–10.08)	32.89 (43.13–20.59)
*Mahonia + Alloberberis*	36.23 (41.36–31.41)	38.67 (44.93–32.89)
*Alloberberis*	33.42 (36.77–31.32)	36.23 (41.36–31.41)
*Mahonia*	28.30 (33.67–23.14)	36.23 (41.36–31.41)
Ranzanieae (*Ranzania*)	N/A	62.24 (72.09–52.00)
Nandinoideae	48.70 (73.76–24.75)	76.54 (88.47–64.26)
Nandineae (*Nandina*)	N/A	48.70 (73.76–24.75)
Leonticeae	26.66 (40.65–12.10)	48.70 (73.76–24.475)
*Caulophyllum*	N/A	26.66 (40.65–12.10)
*Leontice + Gymnospermium*	18.21 (30.94–7.55)	26.66 (40.65–12.10)
*Leontice*	8.04 (17.82–1.27)	18.21 (30.94–7.55)
*Gymnospermium*	10.70 (21.69–2.43)	18.21 (30.94–7.55)
Nandinoideae + Berberidoideae	76.54 (88.47–64.26)	81.57 (93.08–70.71)
Podophylloideae	74.62 (86.50–62.97)	81.57 (93.08–70.71)
Podophylleae	21.61 (32.90–12.07)	43.68 (57.10–28.36)
*Dysosma + Diphylleia*	16.59 (26.19–9.37)	21.61 (32.90–12.07)
*Dysosma*	11.47 (17.94–5.75)	14.08 (23.65–7.70)
*Podophyllum + Sinopodophyllum*	5.15 (12.33–0.49)	21.61 (32.90–12.07)
*Podophyllum*	N/A	5.15 (12.33–0.49)
*Sinopodophyllum*	N/A	5.15 (12.33–0.49)
Achlydeae (*Achlys*)	N/A	43.68 (57.10–28.36)
Bongardieae (*Bongardia)*	N/A	52.09 (66.53–36.22)
Epimedieae	20.93 (29.58–13.02)	65.41 (78.04–53.41)
*Epimedium*	13.43 (19.03–7.84)	20.93 (29.58–13.02)
*Vancouveria*	7.58 (14.72–0.87)	20.93 (29.58–13.02)
Jeffersonieae	23.15 (52.72–3.65)	74.62 (86.50–62.97)
*Jeffersonia*	N/A	23.15 (52.72–3.65)
*Plagiorhegma*	N/A	23.15 (52.72–3.65)

## Discussion

### Variation in Berberidoideae Plastome Structure

Despite the functional importance of chloroplasts in photosynthesis and ostensibly the conserved nature of plastid genomes in both structures and contents (Mower and Vickrey, 2018), IR expansions/contractions have been reported across land plants (Goulding et al., 1996; Zhu et al., 2016). In Berberidaceae, early chloroplast restriction site mapping study (Kim and Jansen, 1994) had revealed IR expansion in *Berberis* and *Mahonia* (including *Alloberberis*). Kim and Jansen’s (1994) observation was attested first by the whole plastome sequence of *M. bealei* (Ma et al., 2013) and subsequent phylogenomic analyses (Sun et al., 2018). In current study, all 23 newly assembled plastomes of *Alloberberis*, *Berberis*, and *Mahonia*, as well as the genus *Moranothamnus* that has never been sampled previously, are featured by significant IR expansions (Supplementary Figure 6), further corroborating previous studies. However, IR expansion was also reported in *R. japonica* (MG234280) by Wang et al. (2018), contradicting to its conspecific plastome sequence MH423072 (Sun et al., 2018) and early chloroplast restriction site mapping study (Kim and Jansen, 1994). Although the inclusion of MG234280 did not affect phylogenetic relationships of *R. japonica* with the rest of Berberidaceae (Supplementary Figure 1), further investigation (e.g., PCR validation) is urgently needed to clarify the SC/IRs junctions in its plastome sequence. Additionally, our analyses also revealed IR expansion and contraction in *E. ecalcaratum* (MN939634) and *E. brevicornu* (MN381716; Zheng et al., 2019), respectively, that have never been reported previously in *Epimedium*. However, in MN803415 (Yao et al., 2020) and MN714008 (Zhang et al., 2020) that are conspecific with MN381716 (Zheng et al., 2019), IR contraction is not detected (Supplementary Figures 6, 9). Further study will be needed to clarify the plastome structure in *E. brevicornu* specifically and *Epimedium* in general.

In addition to IR expansion, substantial length variation in *accD* gene featured by insertions and deletions of repeat sequences was revealed in all sampled Berberidoideae plastomes (Supplementary Figures 7, 8). AccD encodes the β-carboxyl transferase subunit of acetyl-CoA carboxylase (ACCase), which is a functionally essential multi-subunit enzyme in charge of the biosynthesis of fatty acids in plants (Kode et al., 2005) including non-photosynthetic parasitic plants (e.g., Su et al., 2019). However, pseudogenized *accD* has been reported in *Primula sinensis* (Liu T.J. et al., 2016) and *Vaccinium macrocarpon* (Fajardo et al., 2013). Additionally, *accD* has been lost independently from chloroplast genomes and relocated to the nucleus in gymnosperms, i.e., gnetophytes (Sudianto and Chaw, 2019) and *Sciadopitys verticillata* (Li et al., 2016), and multiple angiosperm species of Acoraceae (Goremykin et al., 2005), Campanulaceae (Hong et al., 2017), Fabaceae (Magee et al., 2010), Geraniaceae (Guisinger et al., 2008), Oleaceae (Lee et al., 2007), and Poales (Harris et al., 2013). Despite the extensive length variation, *accD* genes in Berberidoideae appear to be functional as their reading frames are intact without frameshift and the residual sequences at the 3′ end are highly conserved (Supplementary Figure 7). Such length variation characterized by repeat sequences in *accD* has also been reported in the legume species *Medicago truncatula* (Gurdon and Maliga, 2014) and the cupressophytes (Li et al., 2018). Gurdon and Maliga (2014) attributed the intragenic expansion and contraction of *accD* in *M. truncatula* to the presence of repeat sequences that could have triggered replication slippage. Li et al. (2018) also hypothesized that the presence of *accD* repeat sequences could have promoted the acceleration of substitution rate and mediated the rearrangement of plastomes in cupressophytes. Further analyses will be conducted to understand the intriguing *accD* length variation in Berberidoideae.

### Ancient Origins of Berberidaceae Genera

Calibrated by fossils of Menispermaceae from the Turonian and *Alloberberis* and *Mahonia* from the Oligocene, the stem age of Berberidaceae was estimated to be 91.69 Ma (95% HPD: 103.18–79.93 Ma), largely congruent with that estimated by Magallón et al. (2015) at 80.28 Ma (95% HPD: 95.84–68.17 Ma), Li et al. (2019) at 87.4 Ma (95% HPD: 98.9–72.9 Ma), and Ramírez-Barahona et al. (2020) at 101.21 Ma (95% HPD: 117.74–87.28 Ma; constrained calibration of a complete set 238 fossils). However, our estimated crown ages of the three subfamilies are much older than those estimated by Sun et al. (2018) [Berberidoideae: 62.24 (95% HPD: 72.09–52 Ma) vs. ca. 16 Ma (95% HPD: 28–6 Ma); Nandinoideae: 48.70 (95% HPD: 73.76–24.75) vs. ca. 24 Ma (95% HPD: 33–13 Ma); Podophylloideae: 74.62 (95% HPD: 86.50–62.97) vs. ca. 32.5 Ma (95% HPD: 36–27 Ma)], in which the divergence times were estimated by constraining the minimum age of the crown group of Berberidaceae at 33.9 Ma. The disparity of age estimates between Sun et al. (2018) and a majority of studies including current one reflects the dubious application of the *Mahonia* fossil to calibrate a deeper node in the former study, resulting in underestimates of ages within the family (Donoghue and Benton, 2007). Indeed, Sun et al. (2018) adopted Magallón et al.’s (2015) calibration strategy that applied the upper Eocene (33.9 Ma) fossil of *Mahonia* as the minimum crown age of Berberidaceae, apparently underestimating the age for the family. Given this, our results (Table 3 and Figure 5) present a more reliable divergence time estimation of the infrafamilial taxa of Berberidaceae than those of Sun et al. (2018).

Within Berberidaceae, our estimated stem ages of genera range from 5.12 Ma (95% HPD: 13.43–0.34 Ma) in *Podophyllum* and *Sinopodophyllum* to 59.74 Ma (95% HPD: 68.56–51.41 Ma) in *Ranzania* (Table 3 and Figure 5). Except for the former two genera that splitted in the early Pliocene, all genera of Berberidaceae were estimated to have originated prior to the early Miocene. While early Pliocene origins of *Podophyllum* and *Sinopodophyllum* are consistent with Liu et al. (2002; 6.52 ± 1.98 Ma) and Wang et al. (2007; 5.8 ± 0.6 Ma), Yu and Chung (2017) has estimated 20.46 Ma (95% HPD: 34.56–2.66 Ma) and 13.78 Ma (95% HPD: 24.47–1.7 Ma) for the stem ages of *Podophyllum* and *Sinopodophyllum*, respectively.

The late Cretaceous origins of the three subfamilies of Berberidaceae estimated in present study are consistent with recent studies of temperate eudicots (e.g., Hypericaceae, Juglandaceae, and Ranunculaceae) in which major lineage diversification had occurred during the Late Cretaceous and Paleocene (Nürk et al., 2015; He et al., 2021; Zhang et al., 2021). Considering the paleoclimate of the Cretaceous, our dating estimation also suggests that the early lineages of Berberidaceae should have adapted to warmer environments, implying niche shifts experienced by extant species (Folk et al., 2020). Notably, the unusually long branch between stem and crown ages of Epimedieae within Podophylloideae also suggests the occurrence of extinction and/or rapid diversification if the sampling bias is ignored (Antonelli and Sanmartín, 2011). Additionally, while stem ages of most genera within each subfamily were estimated during the Oligocene and Early Miocene, our result suggests the association between the rise of these genera and global climatic deterioration (i.e., temperature cooling and enhanced seasonality) since the Neogene (Smith and Donoghue, 2010). In contrast, the later divergence between *Podophyllum* and the montane *Sinopodophyllum* may be more likely related to the uplift history of the Pan-Himalayan region (Xing and Ree, 2017).

### Conflicts Between Plastome and Nuclear Phylogenies

With the inclusion of *Alloberberis* and *Moranothamnus* and expanded sampling of *Berberis* and *Mahonia*, our plastome phylogenomic analyses support the monophyly of Berberideae, its sister relationship with *Ranzania*, and the monophyly of Berberidoideae, Nandinoideae, and Podophylloideae (Figure 3), corroborating infrafamilial classification in Berberidaceae (Wang et al., 2009; Yu and Chung, 2017; Sun et al., 2018). However, while previous (Sun et al., 2018) and our current plastome trees both place Berberidoideae sister to Nandinoideae (Figure 3), Nandinoideae was resolved as the sister group of Podophylloideae in the combined ML tree of Yu and Chung (2017) and the recently released Kew Tree of Life (KToL) reconstructed using the Hyb-Seq Angiosperms 353 bait set (Johnson et al., 2019; Baker et al., 2021). The conflicting subfamilial relationships of Berberidaceae could have resulted from multiple causes including sampling issues, incomplete lineage sorting (ILS), and hybridization/introgression (Wendel and Doyle, 1998). Given the congruent results between the nuclear trees, i.e., ITS (Yu and Chung, 2017) and the Angiosperms 353 bait set (Baker et al., 2021), the conflicting relationships between plastomes and nuclear datasets observed at the deep level in Berberidaceae seems more likely due to hybridization in the ancient time (Stull et al., 2020).

Within the tribe Berberideae, our phylogenomic analyses (Figure 3) reveal four clades corresponding to *Alloberberis*, *Berberis*, *Mahonia*, and *Moranothamnus*, supporting Yu and Chung’s (2017) classification. However, relationships of the four genera differ between the plastome and the nrDNA phylogenies (Figure 4), as well as Yu and Chung (2017). Specifically, while *Mahonia* and *Alloberberis* are placed in one clade sister to clade *Berberis* + *Moranothamnus* in the plastome tree, in the nrDNA tree *Alloberberis* and *Moranothamnus* formed a strongly supported clade (UFBS: 96) sister to *Berberis*, with *Mahonia* further sister to the clade *Berberis* + *Alloberberis* + *Moranothamnus* (Figure 4). Although ILS could have led to this phylogenetic incongruence (Wendel and Doyle, 1998), the conflicting relationships between plastome and nrDNA tree can also be explained by hybridization between *Berberis* and *Mahonia* (García et al., 2017). Under this scenario, *Berberis* and *Mahonia* should be the maternal parents for *Moranothamnus* and *Alloberberis* (Figure 3), respectively, given cytoplasmic DNA is known to be maternally inherited in Berberidaceae (Zhang et al., 2003). Coupled with the ancient splits of the four genera (Figure 5), *Alloberberis* and *Moranothamnus* could have resulted from ancient reciprocal hybridization (Popelka et al., 2019) between *Berberis* and *Mahonia* preceding subsequent radiations of the two parental genera (García et al., 2017). Additionally, the hybrid origins of *Alloberberis* and *Moranothamnus* could also explain their combined morphology and more restricted geographic distributions relative to *Berberis* and *Mahonia* (Yu and Chung, 2017). Given that *Alloberberis* and *Moranothamnus* are both distributed in western North America, the ancestral ranges of *Berberis* and *Mahonia* are likely also in the New World, as suggested in recent biogeographic study (Chen et al., 2020). Because contemporary intergeneric hybrids between *Berberis* and *Mahonia* (×*Mahoberberis*) rarely occur naturally (Ahrendt, 1961; Rounsaville and Ranney, 2010), the proposition on the hybrid origins of *Alloberberis* and *Moranothamnus* implies a weaker reproductive isolation between the two parental genera in the ancient time.

Within Podophylloideae, although relationships among the five major clades (Figure 3A) are largely congruent with previous studies (Wang et al., 2007; Sun et al., 2018), substantial conflicts exist within tribe Podophylleae between current and previous studies. First, in current and four previous studies (Wang et al., 2007; Sun et al., 2018; He et al., 2019; Li and Dong, 2020), *Sinopodophyllum* is placed sister to *Podophyllum*; however, *Sinopodophyllum* was resolved as sister to *Diphylleia* + *Dysosma* + *Podophyllum* in Ye et al. (2018) and *Dysosma* + *Diphylleia* in Yu and Chung (2017). Second, while our plastome tree resolves *Diphylleia* as a paraphyletic grade sister to *Dysosma*, *Dysosma* was resolved sister to *Diphylleia* + *Podophyllum* in Ye et al. (2018), paraphyletic grade sister to *Diphylleia* + *Podophyllum* + *Sinopodophyllum* in He et al. (2019) and Li and Dong (2020), and polyphyletic in Mao et al. (2016). Third, while all three samples species of *Diphylleia* form a clade in Ye et al. (2018), He et al. (2019), and Li and Dong (2020), the genus is paraphyletic in Mao et al. (2016) and current study (Figure 3A). One important issue that could have contributed to the conflicting results is the very different strategies utilized to analyze the plastome sequences. In Sun et al. (2018) and Ye et al. (2018), only protein-coding genes (CDS) were analyzed, while He et al. (2019) and Li and Dong (2020) used whole plastomes for phylogenetic reconstruction. Because rates of molecular evolution are in general slower in woody species than the herbaceous members of the same taxonomic group (Smith and Donoghue, 2008; Smith and Beaulieu, 2009), we used the full plastome sequences specifically to increase phylogenetic resolution within Berberidoideae. Another factor that might lead to conflicting relationships is partitioning (Kainer and Lanfear, 2015). While no information regarding partitioning were reported in Ye et al. (2018), He et al. (2019), and Li and Dong (2020), we partitioned the plastome sequences into CDS, RNA regions, introns, and IGS, with CDS further partitioned into three parts by codon positions, to take into account rate variation. Additionally, while the monophyly of *Diphylleia* was supported by a combined tree of cpDNA (*matK* and *rbcL*) and ITS2 (Wang et al., 2007) and ITS (Mao et al., 2014), *matK* and *rbcL* alone did not provide enough phylogenetic information for the monophyly of *Diphylleia* in Wang et al. (2007). Interestingly, Bayesian phylogenetic analysis of *CYP719A*, a podophyllotoxin biosynthesis gene that could have experienced relaxed purifying selection, showed that both *Diphylleia* and *Dysosma* are not monophyletic (Mao et al., 2016). These conflicting relationships within Podophylleae again could have resulted from ILS and/or hybridization.

To examine whether ILS or hybridization has contributed to conflicting phylogenetic relationships between plastome and nuclear phylogenies, a robust species tree reconstructed from multi-locus genome data (Morales-Briones et al., 2018) such as Angiosperm 353 bait set (Johnson et al., 2019) could provide a promising solution to resolve conflict phylogenetic relationships between plastome and nuclear genes (Shee et al., 2020).

### Infrafamilial Classification of Berberidaceae

Based on the robust (Figure 3) and dated phylogenomic relationships (Figure 5) reconstructed using completed generic sampling of plastome sequences of Berberidaceae, we evaluate different generic concepts outlined in Supplementary Table 1 using criteria advocated by Backlund and Bremer (1998), Linder et al. (2010), and Heenan and Smissen (2013). Accordingly, our current plastome phylogenomic study (Figures 3, 5) corroborates the classification of four genera within Berberideae (Yu and Chung, 2017). Within Podophylleae, although *Diphylleia* is paraphyletic in our plastome tree (Figure 3), the apparent morphological (Figure 1M), ecological, phytochemical, anatomical, cytological, and palynological coherence of the genus (Ying et al., 1984; Stearn, 2002) and monophyly as revealed by ITS trees (Wang et al., 2007; Mao et al., 2014) also favor the generic status of this long-recognized genus, though hybridization probably also had occurred in the past. We also support the generic status of the EA *Sinopodophyllum* given its morphological (Figure 1L), geographic, and evolutionary distinctness (Figure 5) from the ENA *Podophyllum* (Ying, 1979). As a member of the earliest diversified clade (i.e., Jeffersonieae) sister to the rest of Podophylloideae, the generic status of *Plagiorhegma* should also be maintained given its early Miocene split from *Jeffersonia* (Figure 5) and morphological (Figure 1S) and geographic uniqueness (Hutchinson, 1920).

The maintenance of the generic status of *Alloberberis*, *Mahonia*, *Moranothamnus*, *Plagiorhegma*, and *Sinopodophyllum* that are often synonymized (Supplementary Table 1) not only acknowledges their morphological, ecological, and evolutionary distinctness, but also underscores the critical conservation status of these genera. Since the 17th century, *Berberis* has been a major target for eradication around the world because barberry species (and a few species of *Mahonia*) are alternative hosts of rust fungi (Peterson, 2018; Barnes et al., 2020). However, *Alloberberis* (Breckenridge, 1983; Harms, 2007), *Moranothamnus* (Moran, 1982), and a majority of Asian *Mahonia* are highly endangered threatened by habitat destruction and overexploitation (Boufford, 2013) for traditional Chinese medicines (He and Mu, 2015). Subsuming *Alloberberis*, *Mahonia*, and *Moranothamnus* under a broadly defined *Berberis s.l.* would likely further exacerbate their critical conservation status given the stereotypical impression of *Berberis* as agricultural weeds. Additionally, because both *P. dubium* (Lee et al., 2018) and *S. hexandrum* (Liu W. et al., 2016) are also rare and exploited for traditional medicines, recognizing and elevating these two distinct species to the generic rank also confers an effective conservation strategy.

Throughout the taxonomic history of Berberidaceae, several tribes (Janchen, 1949; Terabayashi, 1985b; Loconte, 1993; Takhtajan, 1997; Wu et al., 2003) had been proposed; however, tribal classification has not been implemented under a molecular phylogenetic context. Based on our phylogenomic analyses (Figures 3, 5), we propose to recognize nine clades as tribes within Berberidaceae. We consulted Reveal’s (1955–onward) “Indices Nominum Supragenericorum Plantarum Vascularium” for priority of the tribal names. Within Berberidoideae, we follow Terabayashi (1985b) and Wu et al. (2003), recognizing tribes Berberideae (including *Alloberberis*, *Berberis*, *Mahonia*, and *Moranothamnus*) and Ranzanieae (including *Ranzania*). Within Nandinoideae, tribes Leonticeae (including *Caulophyllum*, *Gymnospermium*, and *Leontice*) and Nandineae (including *Nandina*) have long been recognized (Supplementary Table 1) and thus are followed here. These two tribes are also characterized by chromosome numbers *x* = 8 and *x* = 10, respectively. Within Podophylloideae, we propose to recognize the five distinct and long-branched clades as tribes (Figures 3, 5). However, while the names Achlydeae, Epimedieae, and Podophylleae are available, the designation Bongardieae (Takhtajan, 1997) was not validly published according to the *Code* (Turland et al., 2018) and the clade *Jeffersonia* + *Plagiorhegma* has never been named. We provide a description for the valid publication of Bongardieae and propose the tribe Jeffersonieae for the latter clade.

### Key to Subfamilies, Tribes, and Genera of Berberidaceae

1. Stamens sensitive; pollen exine psilate and imperforate……………………………………………….2 (Berberidoideae)
1. Stamens not sensitive; pollen exine sculptured and perforate………………………………………………………………………………6
2. Herbaceous……………………………………….Ranzanieae (*Ranzania*)
2. Woody…………………………………………………………. 3 (Berberideae)
3. Stem dimorphic………………………………………………………………….. 4
3. Stem monomorphic……………………………………………………………. 5
4. Stem spineless; leaves 3–9-foliolate…………………….. *Alloberberis*
4. Stem almost always spiny; leaves unifoliolate…………….*Berberis*
5. Leaves imparipinnate, 5–40-foliolate………………………. *Mahonia*
5. Leaves uni- to 7-foliolate………………………………*Moranothamnus*
6. Chromosome base number *x* = 8 or 10……… 7 (Nandinoideae)
6. Chromosome base number *x* = 6………… 10 (Podophylloideae)
7. Woody……………………………………………….. Nandineae (*Nandina*)
7. Herbaceous…………………………………………………….. 8 (Leonticeae)
8. Rhizomatous; inflorescence cymose, bracts subulate; flowers calyculate………………………………………………………….*Caulophyllum*
8. Tuberous; inflorescence a raceme or panicle; bracts foliaceous; flowers excalyculate…………………………………………………………….. 9
9. Leaf solitary, stipulate; seeds exposed by papery pericarp…………………………………………………….. *Gymnospermium*
9. Leaves 2–4, sheathing, seeds enclosed in an inflated bladder………………………………………………………………………*Leontice*
10. Perianth abscent ………………………………………Achlydeae (*Achlys*)
10. Perianth present……………………………………………………………….. 11
11. Leaves pinnate, with more than six pinnae ……………………………………………………….Bongardieae (*Bongardia*)
11. Leaves simple, lobed, or ternately compound……………………. 12
12. Nectaries absent; aril present…………………………………………….. 13
12. Nectaries present; aril absent …………………….16 (Podophylleae)
13. Evergreen, petiolules presence, multicellular leaf pubescence present; more than one flowers in an inflorescence………………………………………………..14 (Epimedieae)
13. Deciduous petiolules absence, multicellular leaf pubescence abscent; one flower in an inflorescence……………………………………………….15 (Jeffersonieae)
14. Leaves cauline and basal, margins spinose; flowers 2-merous, stamens 4……………………………………………………………..*Epimedium*
14. Leaves basal, margins not spinose; flowers 3-merous, stamens 6……………………………………………………………………….. *Vancouveria*
15. Leaves compound; stamens 8……………………………….. *Jeffersonia*
15. Leaves simple; stamens 6…………………………………. *Plagiorhegma*
16. All leaves with petiole attached to the leaf base…………………………………………………………… *Sinopodophyllum*
16. All leaves peltate……………………………………………………………….. 17
17. Anther dehiscence valvate; ovule anatropous……….. *Diphylleia*
17. Anther dehiscence longitudinal; ovule hemitropous…………. 18
18. Flowers several in fascicle; stamens 6………………………. *Dysosma*
18. Flowers solitary; stamens more than 8………………. *Podophyllum*

### Conspectus of the Infrafamilial Classification of Berberidaceae

Subfamily Berberidoideae Eaton (1836)

   Tribe Berberideae Rchb. (1832)

      *Alloberberis* C.C.Yu & K.F.Chung, *Berberis* L., *Mahonia* Nutt., *Moranothamnus* C.C.Yu & K.F.Chung

   Tribe Ranzanieae Kumaz. ex Terab. (1985)

      *Ranzania* T.Ito

Subfamily Nandinoideae Heintze (1927)

   Tribe Leonticeae (Spach) Kosenko (1980)

      *Caulophyllum* Michx., *Gymnospermium* Spach, *Leontice* L.

   Tribe Nandineae Bernh. (1833)

      *Nandina* Thunb.

Subfamily Podophylloideae Eaton (1836)

   Tribe Achlydeae Bernh. (1833)

      *Achlys* DC.

Tribe Bongardieae Takht. ex C.L.Hsieh, C.C.Yu & K.F.Chung, **tr. nov.**

      *Bongardia* C.A.Mey.

   Tribe Epimedieae Dumort. (1829)

      *Epimedium* L., *Vancouveria* C.Morren & Decne.

   Tribe Jeffersonieae C.L.Hsieh, C.C.Yu & K.F.Chung, **tr. nov.**

      *Jeffersonia* Barton, *Plagiorhegma* Maxim.

Tribe Podophylleae DC. (1817)

      *Diphylleia* Michx., *Dysosma* Woodson, *Podophyllum* L., *Sinopodophyllum* T.S.Ying

Tribe **Bongardieae** Takht. ex C.L.Hsieh, C.C.Yu & K.F.Chung, **tr. nov.** – Type: *Bongardia* C.A.Mey.

Bongardieae Takht., Diversity and Classification of Flowering Plants 91. 1997, *num. nud*.

*Diagnosis*. – Perennial herbs, tuberous. *Tuber* subglobose. *Leaves* glabrous, somewhat fleshy, petiolate, imparipinnate with 7–17 leaflets; leaflets sometimes in whorls of 3 or 4, sessile, obovate to oblong, glaucous-green, usually coarsely toothed from the tip. *Inflorescence* a loose panicle with long scape, 20–60 cm tall. *Flowers* long-stalked; sepals 6, concave, suborbicular or ovate, caducous; petals 6, yellow, oblong–ovate, lanceolate or elliptic-oblong, tips sometimes irregularly crenate. *Stamens* 6. *Ovary* with 5–6 basal ovules, ovoid. *Fruit* a capsule, ovoid, papery, opening from the top by short, acute valves; seeds 1–4, black, pruinose.

*Accepted genus*. – This tribe contains one genus *Bongardia* C.A.Mey., which is distributed from southern Greece, northern Africa, Middle East to as far east as Pakistan.

*Note*. – As far as we can track, Bongardieae was first seen in Takhtajan (1997), reiterated in Takhtajan (2009), and adopted by Wu et al. (2003) and Lu and Tang (2020). However, when Takhtajan (1997) published Bongardieae, he did not provide a clear indication of the rank (*Code* Article 37.1), a description/diagnosis (*Code* Article 38.1) in Latin (*Code* Article 39.1), nor a type designation (*Code* Article 40.1). Consequently, Bongardieae Takht. (1997) was not validly published and thus the designation is a *nomen nudum* (Turland et al., 2018).

Tribe **Jeffersonieae** C.L.Hsieh, C.C.Yu & K.F.Chung, **tr. nov.** – Type: *Jeffersonia* Barton

*Diagnosis*. – Perennial herbs, rhizomatous, deciduous. *Rhizome* short, slender; aerial stems absent. *Leaves* basal; petiole long, slender; leaf blade suborbicular or reniform-orbicular in overall outline, simple or divided into 2 sessile leaflets, palmately veined, margin entire or shallowly lobed. *Flowers* scapose, solitary. *Sepals* 3 or 4, caducous. *Petals* 6 or 8, obovate, pale-purple or white. *Stamens* 6 or 8, antipetalous. *Ovary* with many ovules, placentation marginal. *Fruit* a capsule, dehiscing transversely or longitudinally; seeds numerous.

*Accepted genera*. – This tribe contains two monotypic genera, *Jeffersonia* Barton and *Plagiorhegma* Maxim., which are disjunctly distributed in eastern North America and East Asia (northeastern China, South Korea, and Russia along Amur River), respectively.

## Data Availability Statement

The datasets presented in this study can be found in online repositories. The names of the repository/repositories and accession number(s) can be found in Supplementary Table 2.

## Author Contributions

K-FC designed the research and provided the research resources. C-CY, Y-LH, and K-FC collected plant materials. Y-LH collected the genomic data. C-LH and Y-LH assembled the plastomes. C-LH analyzed the data and prepared the figures and tables. C-LH, C-CY, and K-FC wrote the manuscript. All authors read and confirmed the manuscript.

## Conflict of Interest

The authors declare that the research was conducted in the absence of any commercial or financial relationships that could be construed as a potential conflict of interest.

## Publisher’s Note

All claims expressed in this article are solely those of the authors and do not necessarily represent those of their affiliated organizations, or those of the publisher, the editors and the reviewers. Any product that may be evaluated in this article, or claim that may be made by its manufacturer, is not guaranteed or endorsed by the publisher.

## Footnotes

1
tree.bio.ed.ac.uk/software/figtree/
