# Supplementary material for: Mahonia vs. Berberis Unloaded: Generic Delimitation and Infrafamilial Classification of Berberidaceae Based on Plastid Phylogenomics
Source: Front Plant Sci. 2022 Jan 6;12:720171. doi: 10.3389/fpls.2021.720171 (PMC8770955; doi:10.3389/fpls.2021.720171)
Supplement: Supplementary file 2 [file Data_Sheet_1.pdf]

Supplementary Table 1. Comparison of different generic concepts of Berberidaceae since 2009. All databases were accessed on 5 May 2021.

<table>
<tr><th>Yu and Chung (2017), Wikipedia[2], Catalogue of Life[3], and present study</th><th>Sun et al. (2018)</th><th>Wang et al. (2007), Wikispecies[4], TPL[5], and WFO[6]</th><th>Wang et al. (2009)</th><th>Cole and Bachelier (2019)</th><th>Christenhusz et al. (2017)[7]</th><th>Chinese Wikipedia[8]</th><th>Angiosperm Phylogeny Website[9]</th><th>Freiberg et al. (2020), LCVP</th><th>Plant of the World online[10]</th></tr>
<tr><td>[1]N = 19</td><td>N = 18</td><td>N = 17</td><td>N = 16</td><td>N = 16</td><td>N = 15</td><td>N = 15</td><td>N = 14</td><td>N = 13</td><td>N = 13</td></tr>
<tr><td colspan="10">Berberidoideae</td></tr>
<tr><td>Berberis</td><td>✓</td><td rowspan="2">Berberis</td><td rowspan="2">Berberis</td><td rowspan="4">Berberis</td><td rowspan="4">Berberis</td><td rowspan="2">Berberis</td><td rowspan="4">Berberis</td><td rowspan="4">Berberis</td><td rowspan="4">Berberis</td></tr>
<tr><td>Moranothamnus</td><td>✓</td></tr>
<tr><td>Mahonia</td><td>✓</td><td rowspan="2">Mahonia</td><td rowspan="2">Mahonia</td><td rowspan="2">Mahonia</td></tr>
<tr><td>Alloberberis</td><td>✓</td></tr>
<tr><td>Ranzania</td><td>✓</td><td>✓</td><td>✓</td><td>✓</td><td>✓</td><td>✓</td><td>✓</td><td>✓</td><td>✓</td></tr>
<tr><td colspan="10">Nandinoideae</td></tr>
<tr><td>Gymnospermium</td><td>✓</td><td>✓</td><td rowspan="2">Gymnospermium</td><td>✓</td><td>✓</td><td>✓</td><td>✓</td><td>✓</td><td>✓</td></tr>
<tr><td>Leontice</td><td>✓</td><td>✓</td><td>✓</td><td>✓</td><td>✓</td><td>✓</td><td>✓</td><td>✓</td></tr>
<tr><td>Caulophyllum</td><td>✓</td><td>✓</td><td>✓</td><td>✓</td><td>✓</td><td>✓</td><td>✓</td><td>✓</td><td>✓</td></tr>
<tr><td>Nandina</td><td>✓</td><td>✓</td><td>✓</td><td>✓</td><td>✓</td><td>✓</td><td>✓</td><td>✓</td><td>✓</td></tr>
<tr><td colspan="10">Podophylloideae</td></tr>
<tr><td>Podophyllum</td><td>✓</td><td>✓</td><td>✓</td><td>✓</td><td>✓</td><td rowspan="2">Podophyllum</td><td rowspan="2">Podophyllum</td><td rowspan="3">Podophyllum</td><td rowspan="4">Podophyllum</td></tr>
<tr><td>Sinopodophyllum</td><td>✓</td><td>✓</td><td>✓</td><td>✓</td><td>✓</td></tr>
<tr><td>Dysosma</td><td>✓</td><td>✓</td><td>✓</td><td>✓</td><td>✓</td><td>✓</td><td>✓</td></tr>
<tr><td>Diphylleia</td><td>✓</td><td>✓</td><td>✓</td><td>✓</td><td>✓</td><td>✓</td><td>✓</td><td>✓</td></tr>
<tr><td>Achlys</td><td>✓</td><td>✓</td><td>✓</td><td>✓</td><td>✓</td><td>✓</td><td>✓</td><td>✓</td><td>✓</td></tr>
<tr><td>Bongardia</td><td>✓</td><td>✓</td><td>✓</td><td>✓</td><td>✓</td><td>✓</td><td>✓</td><td>✓</td><td>✓</td></tr>
<tr><td>Epimedium</td><td>✓</td><td>✓</td><td>✓</td><td>✓</td><td>✓</td><td>✓</td><td>✓</td><td>✓</td><td>✓</td></tr>
<tr><td>Vancouveria</td><td>✓</td><td>✓</td><td>✓</td><td>✓</td><td>✓</td><td>✓</td><td>✓</td><td>✓</td><td>✓</td></tr>
<tr><td>Jeffersonia</td><td rowspan="2">Jeffersonia</td><td>✓</td><td>✓</td><td>✓</td><td rowspan="2">Jeffersonia</td><td rowspan="2">Jeffersonia</td><td rowspan="2">Jeffersonia</td><td rowspan="2">Jeffersonia</td><td>✓</td></tr>
<tr><td>Plagiorhegma</td><td>✓</td><td>✓</td><td>✓</td><td>✓</td></tr>
</table>

[1]Number of genera (*N*); [2]Wikipedia (https://en.wikipedia.org/); [3]Catalogue of Life (http://www.catalogueoflife.org/); [4]Wikispecies (https://species.wikimedia.org/); [5]The Plant List [TPL; http://www.theplantlist.org/; excluding *Holboellia* (Lardizabalaceae) and *Odostemon* Raf. (synonymy of *Mahonia*)]; [6]World Flora Online [WFO; http://www.worldfloraonline.org/; excluding *Holboellia* (Lardizabalaceae)]; [7]Christenhusz et al. (2017) lists 15 genera in the synopsis but states *N* = 14; [8]Chinese Wikipedia (https://zh.wikipedia.org/);[9]APWeb (Angiosperm Phylogeny Website: http://www.mobot.org/MOBOT/research/APWeb/) [10]Kew Science Plant of the World *online* (http://www.plantsoftheworldonline.org).

Supplementary Table 2. Summary of all plastome sequences sampled in this study. (excel file)

Supplementary Table 3. Gene contents of Berberidaceae plastomes and genes used in phylogenetic analyses.

Function	Gene category	Gene name
Photosynthesis	ATP synthase	*atpA, atpB, atpE, atpF*, atpH, atpI*
	Cytochrome b/f complex	*petA, petB (2)*, petD (2)*, petG, petL, petN*
	NADH dehydrogenase	*ndhA*, ndhB (2)*, ndhC, ndhD, ndhE, ndhF, ndhG, ndhH, ndhI, ndhJ, ndhK*
	Photosystem I	*psaA, psaB, psaC, psaI, psaJ*
	Photosystem II	*psbA, psbB (2), psbC, psbD, psbE, psbF, psbH (2), psbI, psbJ, psbK, psbL, psbM, psbN (2), psbT (2), psbZ*
	Rubisco large subunit	*rbcL*
Self-replication	Ribosomal RNAs (rRNA)	*rrn4.5 (2), rrn5 (2), rrn16 (2), rrn23 (2)*
	Large subunit of ribosome	*rpl2 (2)*, rpl14 (2), rpl16 (2), rpl20, rpl22 (2), rpl23(2), rpl32, rpl33, rpl36 (2)*
	RNA polymerase	~~*rpoA*~~, *rpoB, rpoC1*, rpoC2*
	Small subunit of ribosome	*rps2, rps3 (2), rps4, rps7 (2), rps8 (2), rps11 (2), rps12†*, rps14, rps15, rps16*, rps18, rps19 (2)*
	Transfer RNAs (tRNA)	*trnA-UGC*(2)**, trnC-GCA, trnD-GUC, trnE-UUC, trnF-GAA, trnG-GCC, trnG-UCC*, trnH-GUG, trnI-GAU (2)*, trnK-UUU*, trnL-CAA (2), trnL-UAA*, trnL-UAG, trnM-CAU, trnM-CAU (2), trnfM-CAU, trnN-GUU(2), trnP-UGG, trnQ-UUG, trnR-ACG (2), trnR-UCU, trnS-GCU, trnS-GGA, trnS-UGA, trnT-GGU, trnT-UGU, trnV-GAC (2), trnV-UAC*, trnW-CCA, trnY-GUA*
Other genes	c-type cytochrome synthesis	*ccsA*
	Envelope membrane protein	*cemA*
	Maturase	*matK*
	Protease	*clpP***
	Subunit of acetyl-CoA-carboxylase	*accD*
	Translation initiation factor IF-1	*infA (2)*
Unknown	Hypothetical chloroplast reading frames (ycf)	*ycf1#, ycf2 (2), ycf3**, ycf4, ycf15(2)*

Genes colored in gray are not included in the phylogenetic and subsequent analyses.

Genes in strikethrough are not present in Berberidoideae plastome but present in other Berberidaceae genera.

(2) indicates genes duplicated in IRs.

*indicates genes with one intron.

**indicates genes with two introns.

† The trans-splicing gene, *rps12*, which possesses three exons with two of which duplicated in IRs.

# The partially duplicated gene which is located on the IR/SC boundary.

Supplementary Table 4. The sequence of 33 types of the seemingly repeats found in the *accD* gene of Berberidoideae plastomes.

Type	Nucleotide sequence	Translation	Total number[2]
R1	GACGGTGATTTCGAG	DGDFE	4
R2	GACGGTGATTTCGAC	DGDFD	8
R3	GACGGTGATTCCCAA	DGDSQ	9
R4	GAAGGTGATTTCGAG	EGDFE	37
R4′[1]	GAAGGTGATTTCGAA	EGDFE	6
R5	GAAAGTGATTTCGAA	ESDFE	4
R6	GAAAGTGATTTCGAC	ESDFD	27
R7	GAAGGTGATTTCGAC	EGDFD	1
R8	GAACGTGATTCCCAA	ERDSQ	5
R9	GAACGTGATTTCGAC	ERDFD	5
R10	GAACGTGATTTCGAG	ERDFE	19
R11	GAAGGTGATTCCCAA	EGDSQ	67
R12	GAAAGTGAGTCCCAA	ESESQ	13
R13	GAAGGTGAGTCCCAA	EGESQ	3
R14	GAAGGTGATTTCAAG	EGDFK	2
R15	GAAGGAGATTCCAAA	EGDSK	4
R15′	GAAGGTGATTCCAAA	EGDSK	1
R16	GAAAATGATTTCGAC	ENDFD	24
R17	GAAAATGATTTCGAG	ENDFE	8
R18	GGAAGTGATTTCGAG	GSDFE	4
R19	GAAAGTGATTTCGAG	ESDFE	120
R20	GAAAGTGATTTAGAG	ESDLE	3
R21	GGAAGTGATTCCCAA	GSDSQ	42
R22	GAAAGTGATTCCCAA	ESDSQ	79
R22′	GAAAGTGATTCTCAA	ESDSQ	8
R23	GAAAGTGATCCCCAC	ESDPH	3
R24	GAAAGTGATCCCCAA	ESDPQ	23
R24′	GAAAGTGATCCGCAA	ESDPQ	1
R25	GAAAGGGATTTCTAT	ERDFY	3
R26	GAAAGCGATTCCGAT	ESDSD	2
R27	GGAAGTAATTCCCAA	GSNSQ	7
R28	GAAAGTAATTCCCAA	ESNSQ	20
R29	GAAAGGGATTTCGAG	ERDFE	1
R30	GAAATCACTTTCGAG	EITFE	5
R31	GAAAGTAATTTCGAG	ESNFE	41
R32	GAAAGTGATTCCGAT	ESDSD	3
R33	GAAAGGGATTTCCAT	ERDFH	2

[1]The repeats end with " ′ " symbol are identical in translation to its original types, but only differ in synonymous changes.

[2]The total number of the repeats across the entire alignment of all Berberidoideae plastomes sampled in this study.

Supplementary Table 5. The length of *accD* genes and repeat content in Berberidoideae plastomes sampled in this study.

Species	cp accession	accD length (bp)	# repeat	Repeat content
Alloberberis fremontii	MT335778	1365	15	R8, R9 (2), R11, R17 (2), R19 (3), R20, R22 (3), R23, R28
Alloberberis higginsiae	MT335779	1365	15	R8, R9 (2), R11, R17 (2), R19 (3), R20, R22 (3), R23, R28
Alloberberis trifoliolata	MT335780	1260	8	R8 (3), R16, R19 (2), R20, R23
Berberis amurensis	KM057374	1737	20	R4 (4), R6 (3), R11 (5), R19 (5), R22 (2), R24
Berberis amurensis var. *latifolia*	KM057377	1752	21	R4 (4), R6 (3), R11 (5), R19 (5), R22 (3), R24
Berberis amurensis var. *quelpaertensis*	KM057376	1722	19	R4 (4), R6 (2), R11 (5), R19 (5), R22 (2), R24
Berberis aristata	MK714340	1683	14	R4 (3), R5, R11 (2), R19 (5), R22 (2), R24
Berberis aristata	MN746308	1842	27	R1, R2 (2), R3 (2), R4, R7, R11 (4), R16, R19 (7), R22 (5), R23, R30 (2)
Berberis dictyophylla	MT335782	1527	6	R6 (3), R11, R19, R24
Berberis hayatana	MT335783	1617	12	R2 (2), R3 (2), R16, R19 (4), R22, R24, R30
Berberis kawakamii	MT335784	1677	16	R1, R2 (2), R3 (2), R11, R16, R19 (5), R22 (2), R24, R30
Berberis koreana	KM057375	1842	27	R4 (4), R6 (6), R11 (7), R19 (7), R22 (2), R24
Berberis morrisonensis	MT335785	1587	10	R4, R6 (3), R11 (2), R19 (2), R22, R24
Berberis nantoensis	MT335806	1692	17	R1, R2 (2), R3 (3), R16, R19 (5), R22 (3), R24, R30
Berberis pruinosa	MT335786	1557	8	R4, R11, R17, R19 (3), R22, R24
Berberis saxicola	MT335787	1842	27	R4 (5), R5 (2), R11 (4), R16, R19 (9), R22 (5), R24
Berberis vulgaris	MT335788	1542	7	R4, R6 (2), R11 (2), R19 (2)
Berberis weiningensis	MW018363	1692	17	R4 (3), R6 (2), R11 (4), R19 (5), R22 (2), R24
Mahonia aquifolium	MT335789	1470	17	R5, R6 (2), R10 (2), R12 (5), R16 (3), R19, R22 (2), R29
Mahonia bealei	KF176554	1452	15	R10, R11, R16, R18, R19, R21 (3), R22 (2), R24, R28, R31 (3)
Mahonia bealei	MH795308	1542	21	R10, R11, R16, R18, R19, R21 (5), R22 (2), R24, R28 (3), R31 (5)
Mahonia chochoco	MT335790	1395	13	R9, R10, R11 (2), R12 (2), R14, R17 (2), R19 (2), R22 (2)
Mahonia dictyota	MT335791	1380	12	R6, R10 (2), R11, R12 (3), R16 (2), R19 (3)
Mahonia fortunei	MF188910	1512	19	R10, R16, R21 (2), R22 (2), R24, R27 (4), R28 (3), R31 (5)
Mahonia fortunei	MH795307	1587	24	R10, R16, R21 (7), R22 (2), R24, R28 (6), R31 (6)
Mahonia fortunei	MT335792	1512	19	R10, R16, R21 (3), R22 (4), R24, R27 (2), R28 (3), R31 (4)
Mahonia ganpinensis	MN417307	1437	14	R10, R16, R21 (5), R22 (2), R24, R31 (4)
Mahonia harrisoniana	MT335793	1455	17	R4, R10, R11, R12 (2), R13 (2), R16 (2), R19 (6), R22 (2)
Mahonia japonica	MT335794	1452	15	R6, R10, R11, R18, R19, R21 (4), R22, R24, R28, R31 (3)
Mahonia lanceolata	MT335795	1470	15	R10 (2), R15 (4), R16 (2), R19 (5), R22 (4)
Mahonia nervosa	MT335796	1395	13	R10, R11, R16, R19 (2), R22 (8)
Mahonia oiwakensis	MN735221	1392	11	R10, R16, R19, R21 (3), R22, R24, R28, R31 (2)
Mahonia oiwakensis	MT335797	1452	15	R10, R16, R21 (4), R22 (3), R24, R28, R31 (3)
Mahonia pallida	MT335798	1335	9	R10, R11, R16, R19 (2), R22 (4)
Mahonia tikushiensis	MT335799	1422	13	R6, R10, R11, R18, R19, R21 (4), R24, R31 (3)
Moranothamnus claireae	MT335800	1542	7	R4, R11 (2), R17, R19 (2), R24
Ranzania japonica	MH423072	1641	10	R25 (3), R26 (2), R32 (3), R33 (2)

The number in the parentheses indicates the copy number of the specific type of repeat presented in the sample. Those repeats without

number behind indicate that the individual only contains a single copy of the specific type of repeat. Abbreviations: cp: chloroplast; R: repeat type; # repeat: the total repeat number in the sample (individual).

Supplementary Table 6. Summary of the substitution models and the best partition schemes estimated by ModelFinder under IQ-TREE based on the best BIC scores.

Data Type	Partition	Partition Scheme	Model*	BIC score
**Plastome (ML)**	CDS1	CDS1	TVM+F+R3	172434.7801
	CDS2	CDS2	GTR+F+R2	150501.8386
	CDS3	CDS3	GTR+F+R4	278223.8519
	intron	intron	GTR+F+R3	165787.2440
	RNA	RNA	TIM+F+R2	25334.8822
	spacer	spacer	K3Pu+F+R4	697191.2832
**nrDNA**	ETS<br>ITS1<br>ITS2	ETS+ITS1+ITS2	TVM+F+G4	15355.6015
	NTS	NTS	TPM2+F+G4	7245.4570
	18S	18S	TNe+I	5978.7449
	5.8S<br>26S	5.8S+26S	TN+F+R2	14752.9420
**Plastome (Dating)**	CDS1	CDS1	GTR+F+I+G4	171775.1844
	CDS2	CDS2	GTR+F+I+G4	149450.3832
	CDS3	CDS3	GTR+F+G4	276564.6876
	intron	intron	GTR+F+I+G4	164973.5875
	RNA	RNA	HKY+F+I+G4	25381.11749
	spacer	spacer	GTR+F+G4	690622.5146

[1] The best fit model for each partition in the best partition scheme based on the best BIC score. Abbreviations: ML: Maximum Likelihood; CDS: protein-coding sequence; nrDNA: nuclear ribosomal DNA; ETS: external transcribed spacer; ITS: internal transcribe spacer; IGS: intergenic spacer; BIC: Bayesian inference criterion.

Supplementary Table 7. Summary of nrDNA assemblies of five extra samples retrieved from SRA database.

Species	SRA accession	# total read	read length (bp)	nrDNA length (bp)	av. Cov.	cov. SD	Trimmomatic settings	GetOrganelle settings
Berberis amurensis	SRR8889259	9329548	76	7141	730.3	169	LEADING:25 TRAILING:20 SLIDINGWINDOW:4:20 MINLEN:45	-R 15 -t 10 -w 0.7 -k 37,49,55,61,65,69 -F embplant_nr --reduce-reads-for-coverage inf
Berberis koreana	SRR8889260	10028836	76	7188	222	60.4	LEADING:25 TRAILING:20 SLIDINGWINDOW:4:20 MINLEN:45	-R 15 -t 10 -w 0.7 -k 37,49,55,61,65,69 -F embplant_nr --reduce-reads-for-coverage inf
Berberis weiningensis	SRR13741681	65337718	150	7187	9269.2	1983.7	LEADING:25 TRAILING:25 SLIDINGWINDOW:4:25 CROP:148 MINLEN:100	-R 15 -t 10 -w 0.7 -k 37,65,85,97,115,121,135 -F embplant_nr --reduce-reads-for-coverage inf
Bongardia chrysogonum	SRR14740366	125835522	150	7009	5315.6	3662.8	LEADING:25 TRAILING:30 SLIDINGWINDOW:4:25 CROP:148 MINLEN:100	-R 15 -t 10 -w 0.7 -k 37,65,85,97,115,121,135 -F embplant_nr --reduce-reads-for-coverage inf
Podophyllum peltatum	ERR3525041	73978352	100	7135	594	140.2	LEADING:25 TRAILING:25 SLIDINGWINDOW:4:25 CROP:98 MINLEN:60	-R 15 -t 10 -w 0.7 -k 37,65,85,97 -F embplant_nr --reduce-reads-for-coverage inf

Abbreviations: SRA: Sequence Read Archive; nrDNA: nuclear ribosomal DNA; av. Cov.: average coverage (×); cov. SD (×): coverage standard deviation.

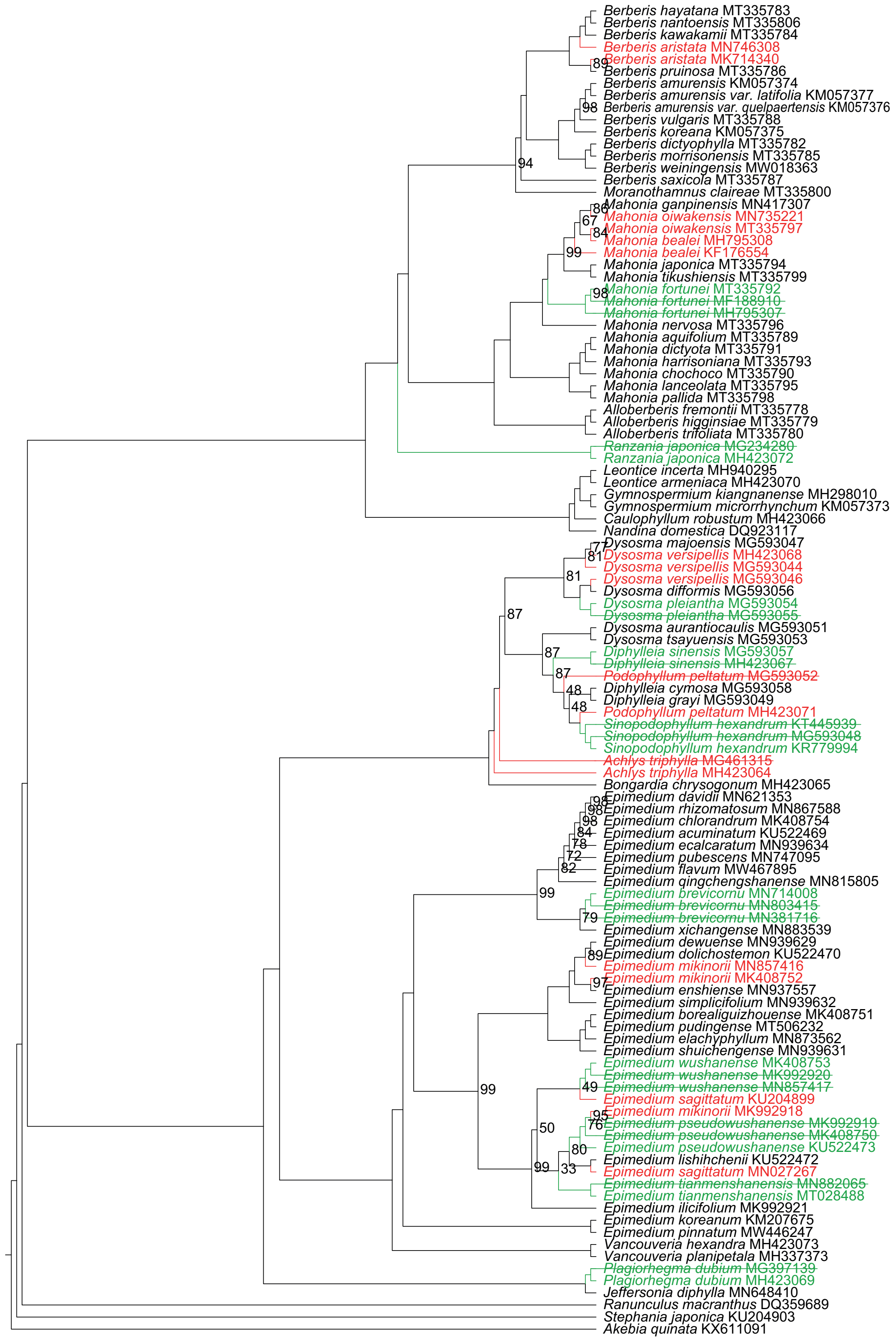

Supplementary Figure 1. A preliminary plastome maximum likelihood cladogram inferred by IQ-TREE on the basis of 110 plastome sequences. Only nodes whose ultrafast bootstrap support (UFBS) values are not 100 were labeled with its exact values. The conspecific samples that form a clade are colored in green, while those are not monophyletic are colored in red. The samples that are not included in the subsequent analyses (maximum likelihood analyses) are struck out.

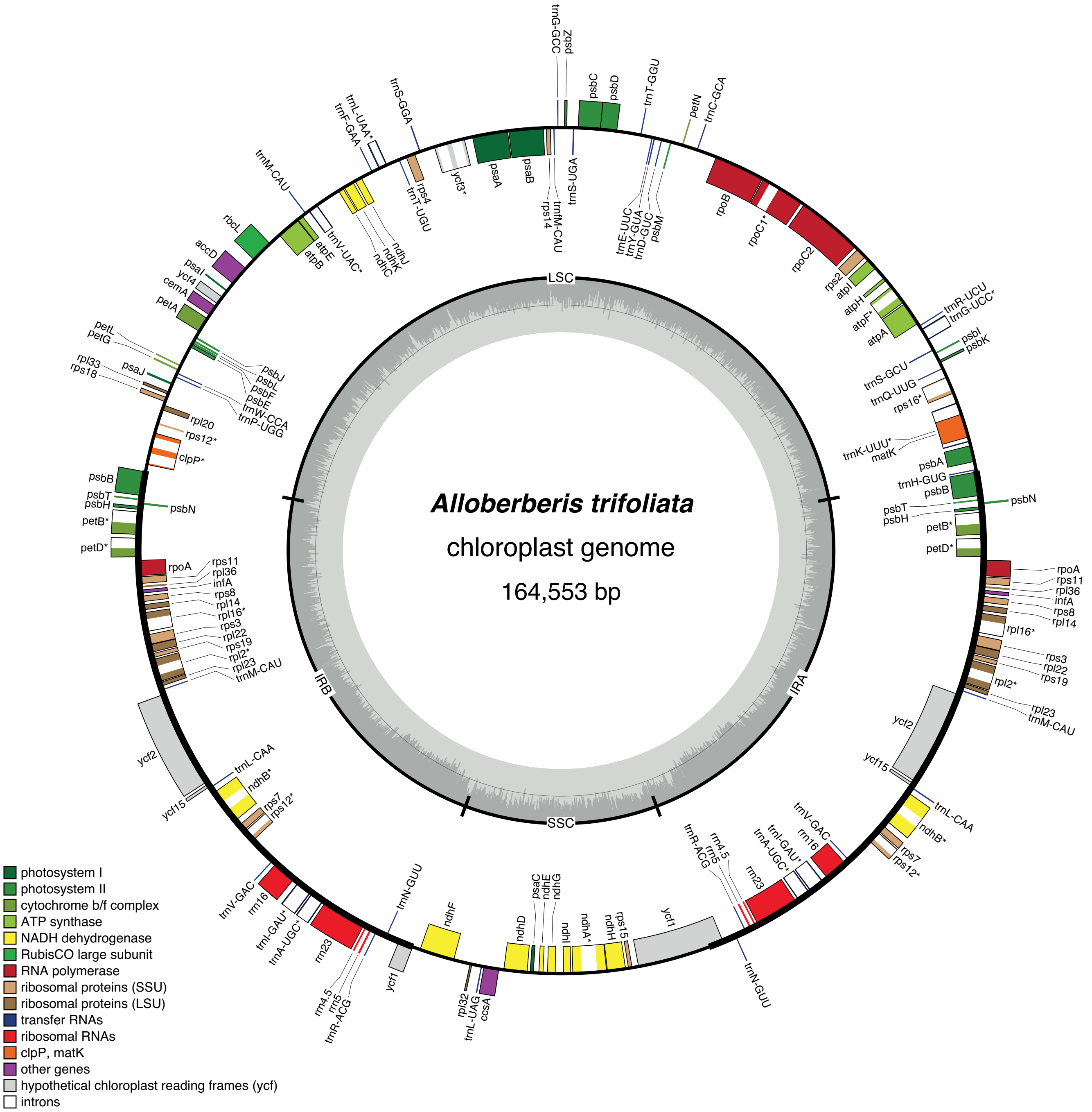

Supplementary Figure 2. Chloroplast genome map of *Alloberberis trifoliolata,* representing the plastome configuration of *Alloberberis*. The genes drawn on the inner side of the outer circle are transcribed clockwise, and those on the outer side are transcribed counterclockwise. IRs are shown in bold lines in the outer circle. The darker gray areas of the inner circle indicate GC contents across the genome with lighter gray areas indicating AT contents. Genes belonging to different functional groups were shown in different color as in the legend.

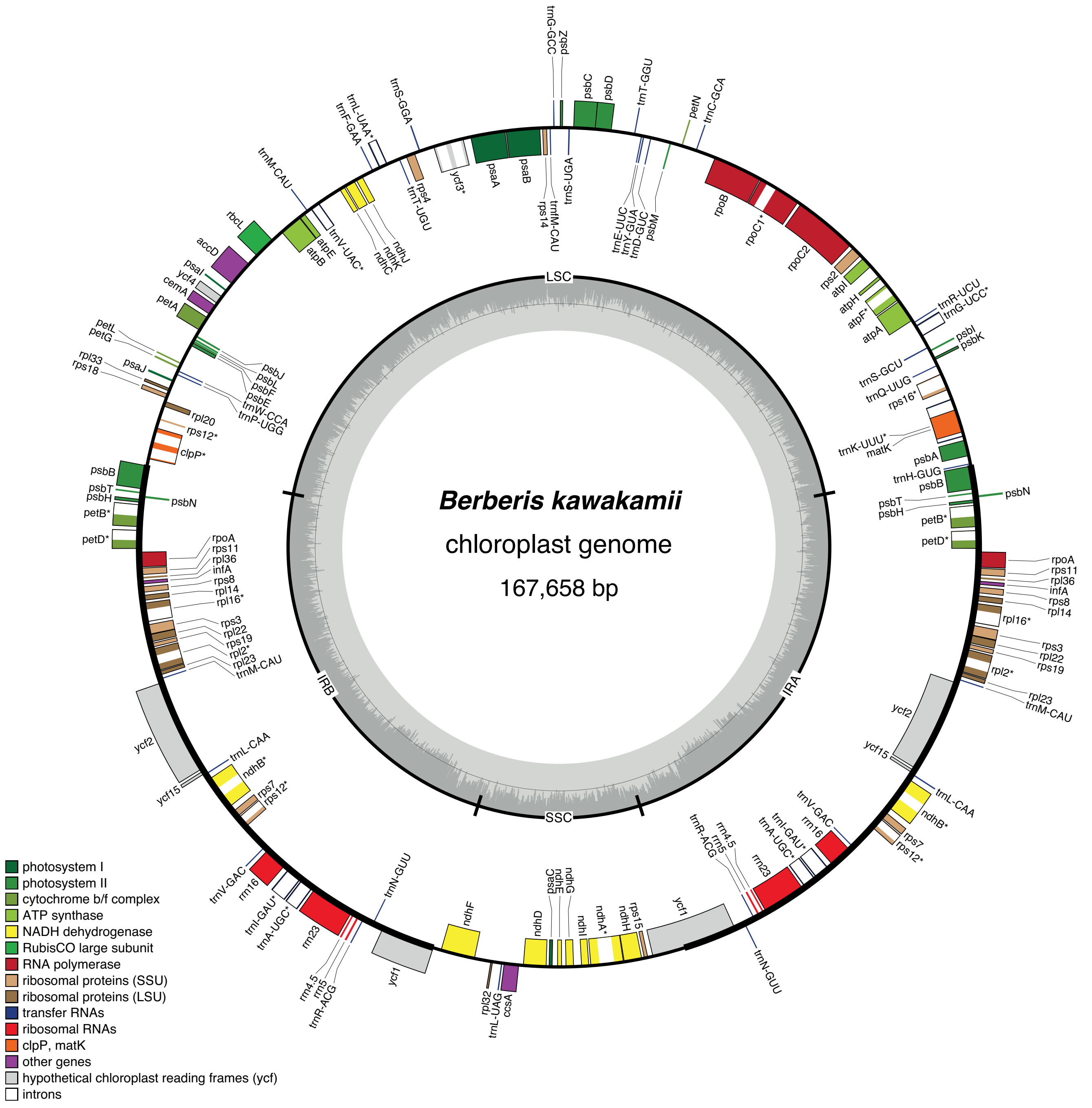

Supplementary Figure 3. Chloroplast genome map of *Berberis kawakamii*, representing the plastome configuration of Taiwanese *Berberis*. The genes drawn on the inner side of the outer circle are transcribed clockwise, and those on the outer side are transcribed counterclockwise. IRs are shown in bold lines in the outer circle. The darker gray areas of the inner circle indicate GC contents across the genome with lighter gray areas indicating AT contents. Genes belonging to different functional groups were shown in different color as in the legend.

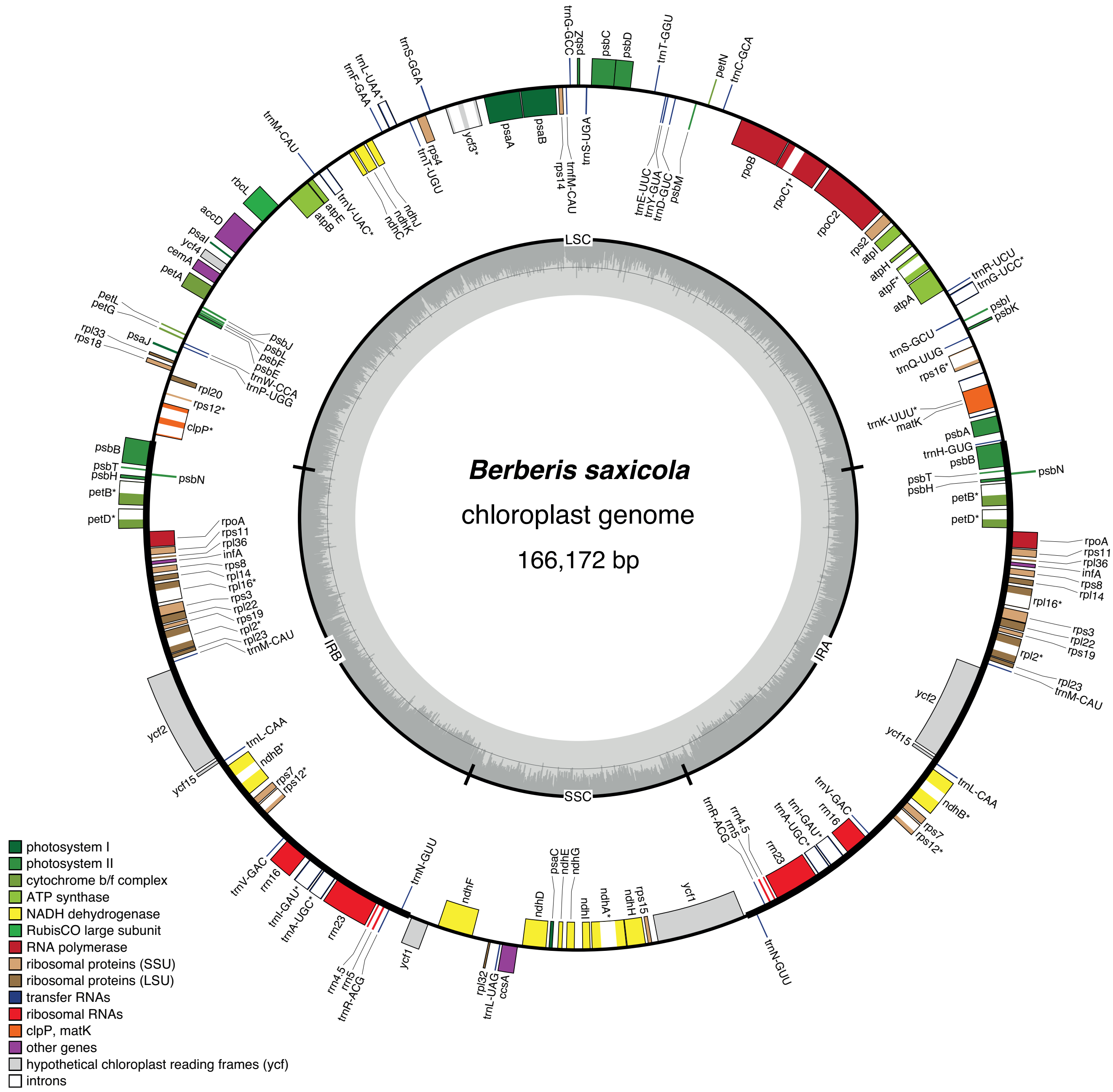

Supplementary Figure 4. Chloroplast genome map of *Berberis saxicola*, representing the plastome configuration of *Berberis*. The genes drawn on the inner side of the outer circle are transcribed clockwise, and those on the outer side are transcribed counterclockwise. IRs are shown in bold lines in the outer circle. The darker gray areas of the inner circle indicate GC contents across the genome with lighter gray areas indicating AT contents. Genes belonging to different functional groups were shown in different color as in the legend.

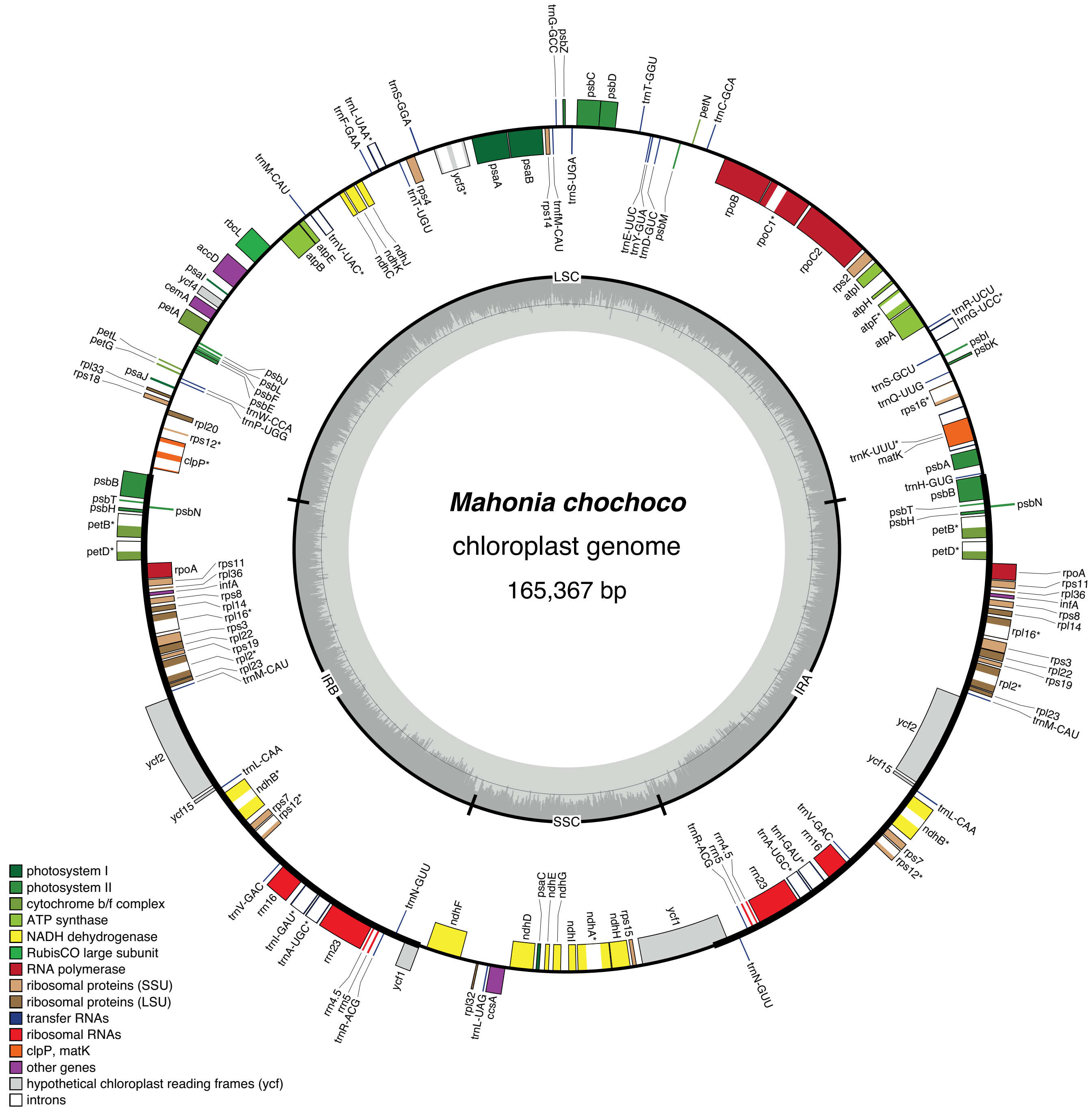

Supplementary Figure 5. Chloroplast genome map of *Mahonia chochoco*, representing the plastome configuration of *Mahonia*. The genes drawn on the inner side of the outer circle are transcribed clockwise, and those on the outer side are transcribed counterclockwise. IRs are shown in bold lines in the outer circle. The darker gray areas of the inner circle indicate GC contents across the genome with lighter gray areas indicating AT contents. Genes belonging to different functional groups were shown in different color as in the legend.

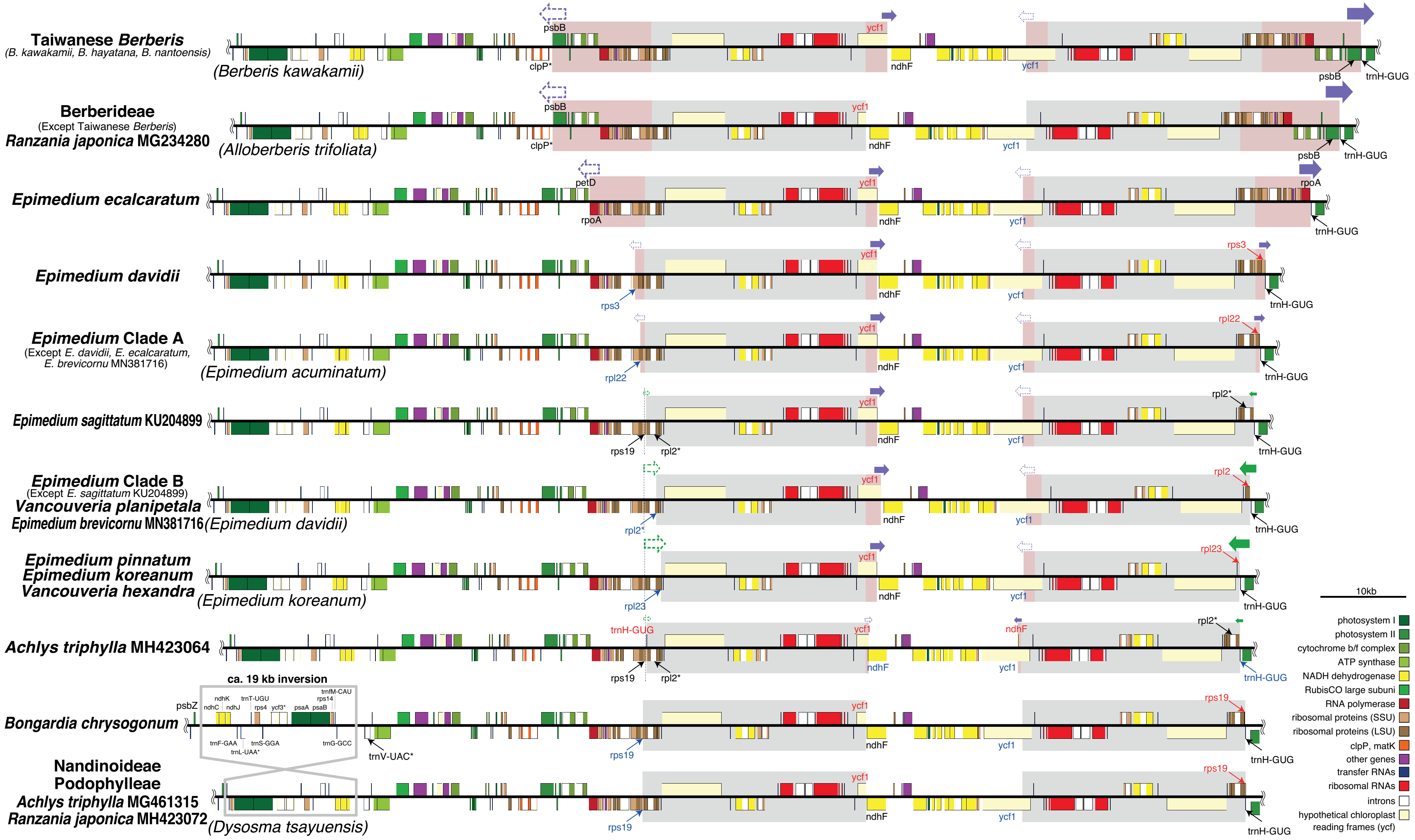

Supplementary Figure 6. IR comparison among plastomes of Berberidaceae species. Note that the genome maps drawn by OGDRAW were linearized and were only partially displayed. Only genes on or beside the IR/SC junctions are labeled. Genes that are located exactly on the IR/SC junctions are colored in blue, while those being partially duplicated by IRs are colored in red. Genes colored in black are neither located on the IR/SC junctions, nor partially duplicated by IRs. Whether IRs (marked by gray boxes) have been expanded (marked by red boxes and purple arrows) or contracted (marked by black dash lines and green arrows) is referred to the boundary configurations that are the

most frequently found across early-diverging eudicots ("subgroup E" in Sun et al., 2016). This type of IR endpoints is also concordant with those in our Nandinoideae & Podophylleae plastomes. The hollow arrows with dashed outlines indicate the original region where the expansion/contraction occurred, while the solid arrows indicate the expanded/contracted areas resulted by IR duplications. The size of the arrows implies the relative length of expansion/contraction. In addition, the ca. 19 kb inversion in the LSC of *Bongardia chrysogonum* plastome is further shown in detail.

(Reference)

Sun, Y., Moore, M. J., Zhang, S., Soltis, P. S., Soltis, D. E., Zhao, T., Meng, A., Li, X., Li, J., & Wang, H. (2016). Phylogenomic and structural analyses of 18 complete plastomes across nearly all families of early-diverging eudicots, including an angiosperm-wide analysis of IR gene content evolution. *Molecular Phylogenetics and Evolution*, 96, pp.93-101.

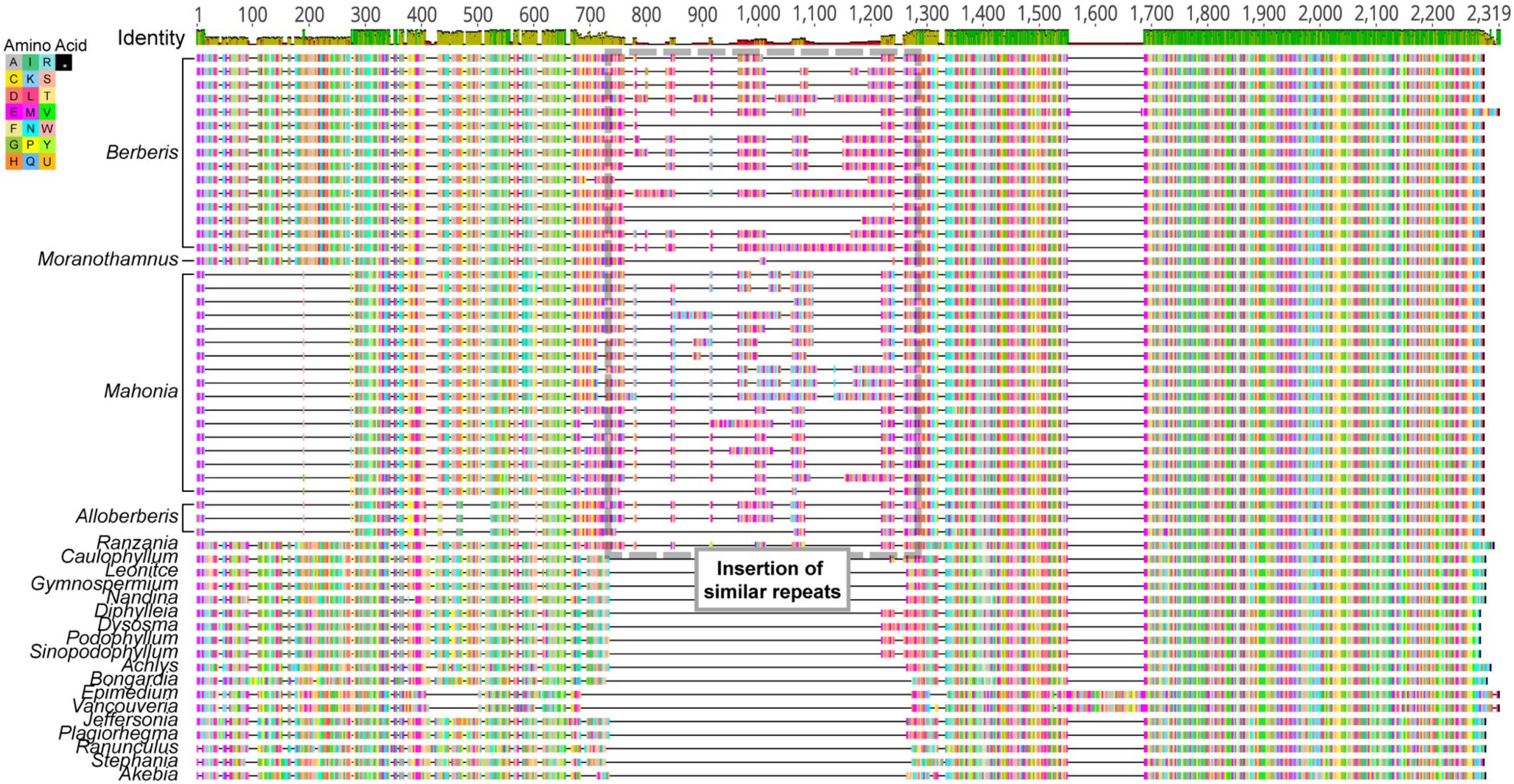

Supplementary Figure 7. The translation alignment of the *acc*D genes of Berberidaceae and three outgroup species. The figure is a screenshot taken from Geneious Prime. Gaps are shown as black lines.

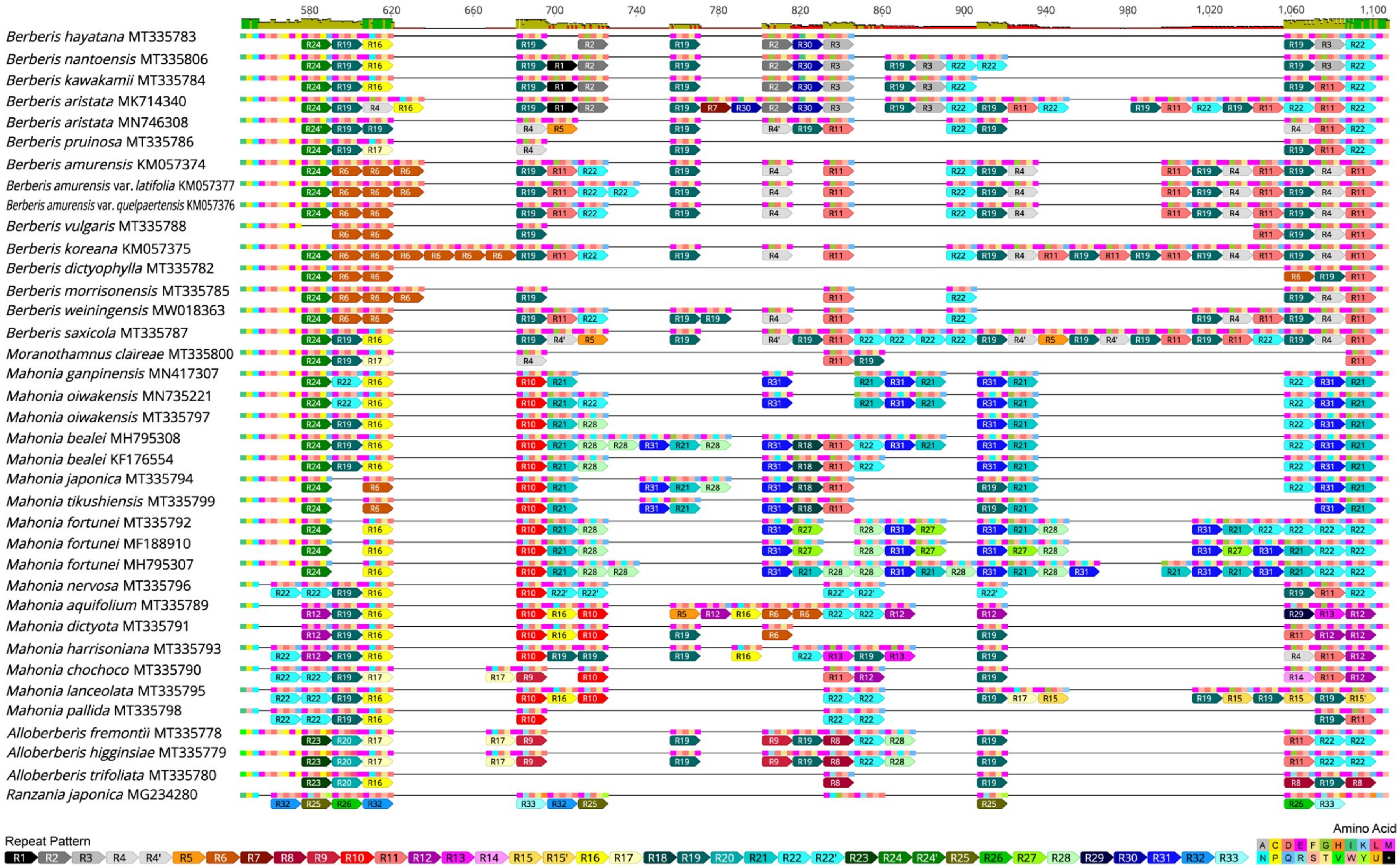

Supplementary Figure 8. The translation alignment of the regions of the seemingly repeated sequences found in Berberidoideae *acc*D genes. The repeats are annotated by different colors based on their types. The figure is a screenshot taken from Geneious Prime. Gaps are shown as black lines.

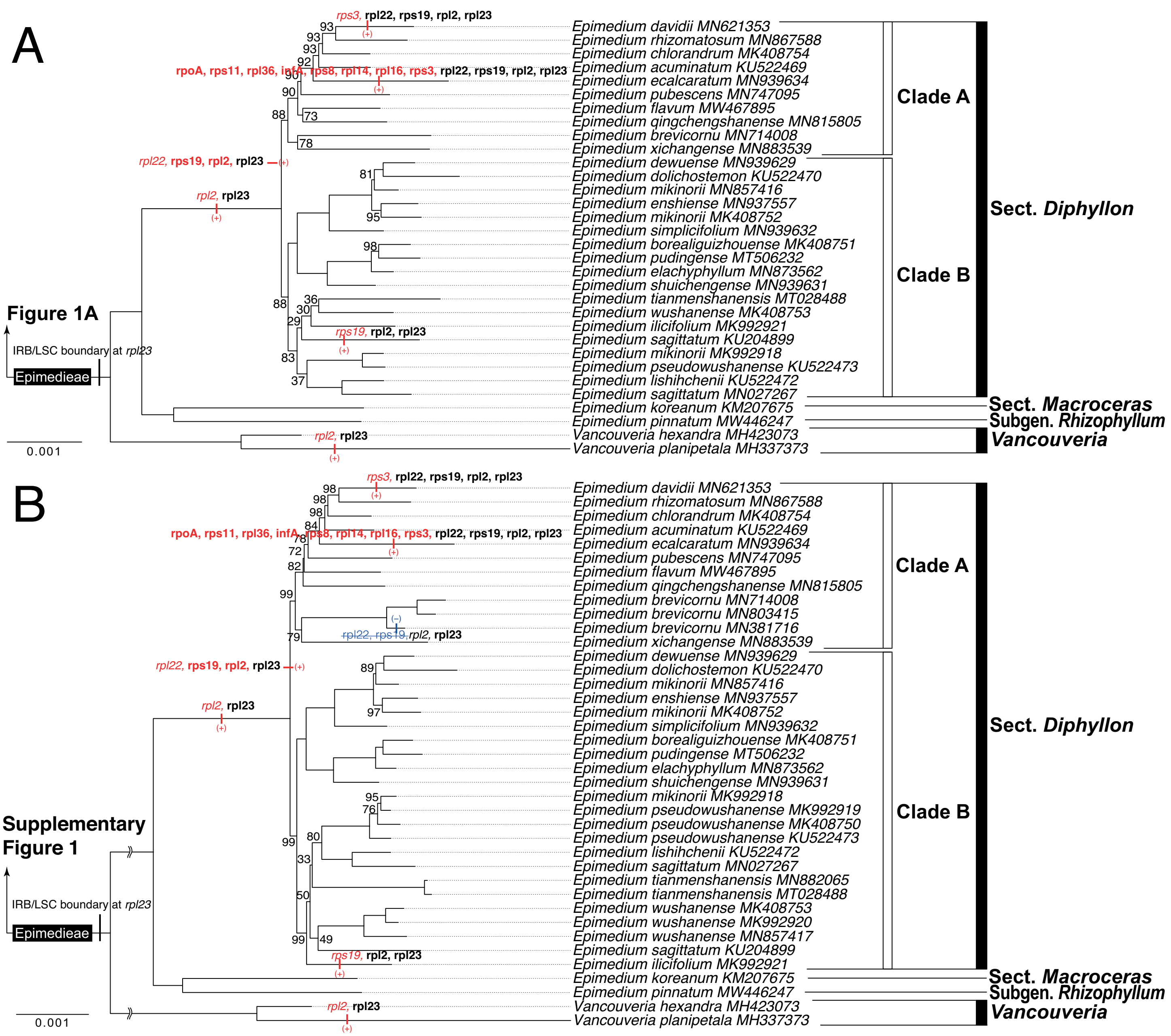

Supplementary Figure 9. The detailed plastome phylogenies of *Epimedium* and *Vancouveria*, which is extracted from **Figure 3A & Supplementary Figure 1**, and the IRB/LSC boundary shift across the two genera. **(A)** The phylogram extracted from **Figure 3A**, in which the monophyletic and conspecific samples were excluded; **(B)** the phylogram extracted from **Supplementary Figure 1**, in which all the conspecific samples were retained. UFBS values that are not 100 are shown on each node. The supporting branches of the nodes whose UFBS value are over 95 are marked in bold line, those with 70–90 UFBS values are shown in regular width, while those with UFBS values under 70 are shown in dashed lines. The boundary shifts are marked on the branches with red (+) symbol indicating expansions while the blue (–) symbol indicates the contraction comparing to the previous state across the tree. The gene that is located on the boundary (i.e., the gene is partially duplicated by IRs) is shown in italic, those outside the IRs are stuck out, while those inside the IRs are shown in bold. By comparing to the previous state across the phylogeny, genes that is located inside the IRs due to IR expansion are colored in red, while those outside the IRs because of IR contraction are colored in blue.
